# Fatty Acid Profile of Lipid Fractions of Mangalitza (*Sus scrofa domesticus*) from Northern Romania: A GC-MS-PCA Approach

**DOI:** 10.3390/foods10020242

**Published:** 2021-01-26

**Authors:** Cornelia Petroman, Gabriela Popescu, Raymond-Nandy Szakal, Virgil Păunescu, Lavinia P. Drăghia, Gabriel S. Bujancă, Cosmina A. Chirilă, Daniel I. Hădărugă, Loredana Văduva, Nicoleta G. Hădărugă, Ioan Petroman

**Affiliations:** 1 Department II—Economy and Company Financing, Banat’s University of Agricultural Sciences and Veterinary Medicine “King Michael I of Romania” from Timişoara, Calea Aradului 119, 300645 Timişoara, Romania; corneliapetroman@usab-tm.ro; 2 Department of Rural Management and Development, Banat’s University of Agricultural Sciences and Veterinary Medicine “King Michael I of Romania” from Timişoara, Calea Aradului 119, 300645 Timişoara, Romania; gabrielapopescu@usab-tm.ro (G.P.); loredanavaduva@usab-tm.ro (L.V.); ioan_petroman@usab-tm.ro (I.P.); 3 Department of Food Science, Banat’s University of Agricultural Sciences and Veterinary Medicine “King Michael I of Romania” from Timişoara, Calea Aradului 119, 300645 Timişoara, Romania; raymondnalbu@gmail.com (R.-N.S.); cosminachirila@yahoo.com (C.A.C.); 4 Department of Physiology and Immunology, “Victor Babeş” University of Medicine and Pharmacy, Eftimie Murgu Sq. 2, 300041 Timişoara, Romania; vpaunescu@umft.ro (V.P.); draghia_lavinia@yahoo.com (L.P.D.); 5 Centre for Gene and Cellular Therapies in the Treatment of Cancer—OncoGen, Clinical County Hospital of Timişoara, Liviu Rebreanu Blvd. 156, 300736 Timişoara, Romania; 6 Department of Food Control, Banat’s University of Agricultural Sciences and Veterinary Medicine “King Michael I of Romania” from Timişoara, Calea Aradului 119, 300645 Timişoara, Romania; gabrielbujanca@yahoo.com; 7 Department of Applied Chemistry, Organic and Natural Compounds Engineering, Polytechnic University of Timişoara, Carol Telbisz 6, 300001 Timişoara, Romania; daniel.hadaruga@upt.ro

**Keywords:** fatty acid profile, lipid fractions, Mangalitza, *Sus scrofa domesticus*, aldehyde degradation compounds, gas chromatography—mass spectrometry, principal component analysis, GC-MS-PCA coupled technique

## Abstract

Mangalitza pig (*Sus scrofa domesticus*) becomes more popular in European countries. The goal of this study was to evaluate the fatty acid profile of the raw and thermally processed Mangalitza hard fat from Northern Romania. For the first time, the gas chromatography-mass spectrometry-Principal component analysis technique (GC-MS-PCA)—was applied to evaluate the dissimilarity of Mangalitza lipid fractions. Three specific layers of the hard fat of Mangalitza from Northern Romania were subjected to thermal treatment at 130 °C for 30 min. Derivatized samples were analyzed by GC-MS. The highest relative content was obtained for oleic acid (methyl ester) in all hard fat layers (36.1–42.4%), while palmitic acid was found at a half (21.3–24.1%). Vaccenic or elaidic acids (trans) were found at important concentrations of 0.3–4.1% and confirmed by Fourier-transform infrared spectroscopy. These concentrations are consistently higher in thermally processed top and middle lipid layers, even at double values. The GC-MS-PCA coupled technique allows us to classify the unprocessed and processed Mangalitza hard fat specific layers, especially through the relative concentrations of vaccenic/elaidic, palmitic, and stearic acids. Further studies are needed in order to evaluate the level of degradation of various animal fats by the GC-MS-PCA technique.

## 1. Introduction

Mangalitza (*Sus scrofa domesticus*) is a domesticated pig of the wild *Sus scrofa ferus*. It becomes more popular not only in the Balkan countries but in all of Europe. There are few breed types that are grown in these regions, such as the white or blonde strain, red strain, and swallow-belly strain [1,2]. They provide high quality meat and fat, having an intramuscular fat content up to 18.2%. On the other hand, the backfat or hard fat thickness is relatively high (4.2–10.2 cm) with an equilibrated distribution of the fatty acid profile. This product is traditionally consumed in Romania, both as a smoked and salted raw product or thermally prepared. Generally, the highest fatty acid content was observed for monounsaturated fatty acids (MUFAs, more than a half) and saturated fatty acids (SFAs, 33–40%). Polyunsaturated fatty acids (PUFAs) are much less concentrated (<11%) [2].

The fatty acid profile of lipid fractions from various pork parts vary among the growing locations (farming), crossbreeding and gender, feed composition, processing, and storage [3,4,5,6,7,8,9]. A study on the influence of crossbreeding on the fatty acid composition in Large White, Duroc, Pietrain, and Landrace pork lines reveal significant differences among the SFA composition (32.9–35.4%). Almost no differences were observed among pork males and females, where oleic acid was the most concentrated (39.8–40.7%) [3]. The fatty acid composition of the pork fat consistently differs with the feed composition. The ω-3 fatty acid content can increase from 1.2–2% to 8.9–16.1% in the muscle of pigs fed with a 10% flaxseed-based diet [4]. Similar studies have been performed on the fatty acid composition of fresh pork cuts regarding the breeding, feeding, and processing methods [7]. The highest differences have been observed for the PUFAs, especially for linoleic acid, with relative concentrations between 6.9–10.3% in the raw fat of pork loin chop from various Australian regions. On the other hand, the ω-3 fatty acids and conjugated linoleic acid were determined at very low concentrations [7]. The effect of cooking on the fat and cholesterol contents of pork cuts is important [10]. The total fat content increased from 7.03% to 14.3% after cooking by roasting or broiling, especially due to the concentrating effect. The decrease of the pork meat quality due to the change of the fatty acid composition after cooking was also observed.

Fatty acid composition was evaluated through the Suidae mammals. Among these, wild boar and domestic pork meat compositions are influenced by the habitats and feeding facilities [8]. No significant differences were observed for the protein content. On the other hand, palmitoleic acid was more concentrated in the domestic pork, but the highest difference was observed for arachidonic acid. This ω-6 PUFA had a relative concentration of 1.3–2% in the wild boar meat, compared with only 0.05–0.87% in the domestic pork.

The presence of MUFA and PUFA moieties in the pig fat and particularly in the Mangalitza hard fat allows partial degradation during thermal processing in the presence of air. The quality of these fatty products decreases due to the oxidative degradation of the fatty acid moieties, e.g., to the harmful free radicals, epoxy derivatives, aldehydes, and trans fatty acids. Moreover, some volatile compounds, such as aldehydes, cause degradation of the organoleptic characteristics of these products. There are few studies related to the stability and degradation of fatty acids and fatty acid glycerides from the pork fat, with most of them dealing with the variation of the fatty acid profile after cooking. The use of various antioxidants for reducing the degradation level of fatty acids has been studied. The lipid oxidation in raw and cooked minced pork, as well as in pork burgers during storage and the antioxidant effect of various plant extracts and powders have been evaluated through the degradation fatty acid derivatives and metabolomics changes [11,12]. In the pork burgers, the main metabolites related to the lipid oxidation were identified by advanced liquid chromatography coupled with multivariate statistical analysis [12], while the oxylipins in the cooked minced pork were quantified by high performance liquid chromatography coupled with tandem mass spectrometry (HPLC-MS/MS) [11]. The higher content of MUFAs and PUFAs in Mangalitza fats determines their lower oxidative and thermal stability. Oleic acid is the most important fatty acid in such fats, as well as linoleic acid from the PUFAs [9,13]. Due to the high content of oleic acid, Mangalitza pig resembles olive oils or fish oils, such as salmon oil [14]. The relative concentration of oleic acid in various Mangalitza fat parts is in the range of 40–51%. Other MUFAs in Mangalitza fat were palmitoleic acid (up to 6%) and vaccenic or elaidic acids as trans isomers. SFAs are also important in Mangalitza fat, especially palmitic, stearic, and myristic acids. However, their relative concentrations differ with the variety, rearing conditions, feeding, and processing [15,16,17,18,19,20].

Coupled modern physical chemical analyses and multivariate statistical techniques are very useful for classification, prediction, and detection of degradation and adulteration of food. There are many such coupled techniques in the food field, but only few related to fatty acid profile of pork. They are related to the classification of conventional, free range, and organic pork meat using FAMEs (fatty acid methyl esters), non-volatile compounds, and volatile compounds composition by chemometric methods (principal component analysis, PCA) [21]. Other study coupled triacylglycerol profiling with DNA analysis and further with PCA and PLS-DA (partial least squares discriminant analysis) in order to authenticate beef, pork, and chicken meat and meat products [22]. According to our knowledge, no such studies have been performed on the Mangalitza fatty acid profile.

The goal of the study was to evaluate the fatty acid profile of unprocessed (raw) and thermally processed lipid fractions of Mangalitza growing in Northern Romania, as well as the level of degradation of unsaturated fatty acid glycerides to the harmful trans isomers or to aldehydes during thermal treatment. For the first time, for such valuable animal products, the similarity/dissimilarity of lipid fractions was also evaluated using a gas chromatography-mass spectrometry coupled with principal component analysis (GC-MS-PCA).

## 2. Materials and Methods

### 2.1. Materials

**Figure 1:**
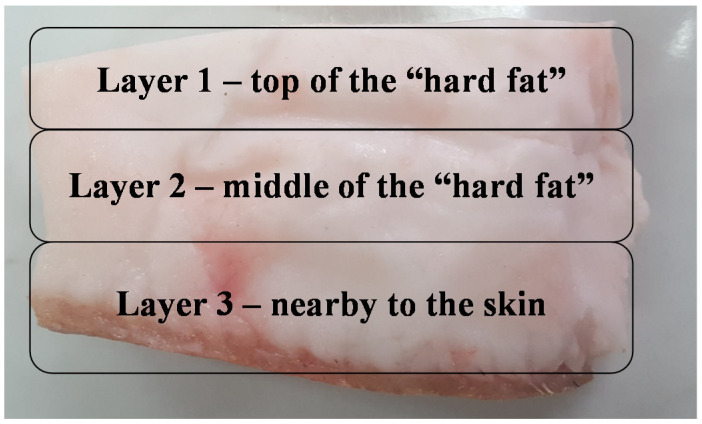
Sampling of the lipid fractions of Mangalitza hard fat.

The fat samples were collected in the autumn of 2019 from mature Mangalitza pigs (*Sus scrofa domesticus*, “blonde” variety, age of 12 months, three male pigs, and weight of 70–80 kg) direct from the producer, which are growing the animals in a traditional and ecological system (Maramures county, Northern Romania, 47°37′5″ N and 23°28′44″ E, altitude ~164 m). In this study, the fatback part (hard fat) has been subjected to analysis, which is one of the most consumed pig fat, usually raw, cured, or fried. Samples have been maintained at 4 °C during the transportation and storage. Three hard fat fractions have been manually separated as following: (1) layer from the top of the hard fat, (2) layer from the middle of the hard fat, and (3) layer nearby to the skin (all of approximately one-third of the total thickness, Figure 1). On the other hand, lipid samples have been subjected or not to thermal processing as following: U-unprocessed (raw) lipid fractions (coded U1, U2, and U3 for all three layers), and P-thermally processed lipid fractions (coded P1, P2, and P3 for all three layers). Thermal processing was performed in a halogen-drying thermo-balance at 130 °C for 30 min, for approximately 5 g of lipid samples (Thermo-balance Partner WPS 210S, Radwag Intelligent Weighing Technology, Inc., capacity 210 g, division 0.001 g/0.01%, 2 halogen lamps/300 W, “strobe” interval 30 s). The European Union (EU) directives and regulations regarding the killing and manipulating of samples have been appropriately considered and respected by the producer, who provided the hard fat samples (Council Regulation (EC) N^o^ 1099/2009 on “The protection of animals at the time of killing,” Directive 2010/63/EU of the European Parliament and of the Council on “The protection of animals used for scientific purposes,” Regulation (EC) N^o^ 853/2004 and N^o^ 854/2004 of the European Parliament and of the Council on “Specific hygiene rules for on the hygiene of foodstuffs, and for the organization of official controls on products of animal origin intended for human consumption,” and Commission Regulation (EC) N^o^ 889/2008 on the “Implementation of Council Regulation (EC) N^o^ 834/2007 on organic production and labelling of organic products with regard to organic production, labelling, and control”). Sample preparation and analysis have been performed in duplicate.

Hexane (GC-grade, Sigma-Aldrich, St. Louis, MO, USA), borontrifluoride-methanol solution (20% BF_3_, Merck & Co., Inc., Kenilworth, NJ, USA) and anhydrous sodium sulphate (p.a., Merck & Co., Inc., Kenilworth, NJ, USA) have been used for the solubilization, derivatization, and drying of the lipid fatty acid glycerides to the corresponding FAMEs. Identification of FAMEs needed a C_8_-C_20_ linear alkane standard mixture (Sigma-Aldrich, St. Louis, MO, USA) and standard FAMEs (FAME37, Sigma-Aldrich, St. Louis, MO, USA).

### 2.2. Derivatization of the Unprocessed and Thermally Processed Lipid Fractions

**Figure 2:**
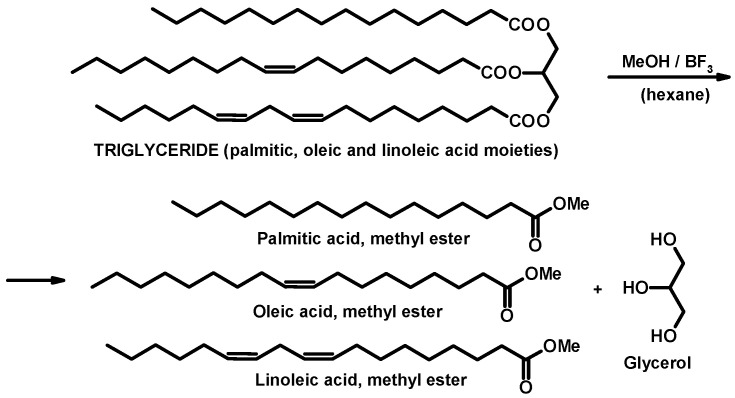
Schematic representation of the derivatization (transesterification) of a fatty acid glyceride (containing palmitic, oleic, and linoleic moieties) to the corresponding palmitic, oleic, and linoleic acid, methyl esters.

Fatty acid profile of the Mangalitza lipid fractions has been obtained after quantitative derivatization (transesterification) of the fatty acid glycerides to the corresponding FAMEs (Figure 2). The derivatization was performed according to the validated method described by Slover and collaborators [10] by borontrifluoride-methanol method, with slight modifications. Shortly, the derivatization was performed in a 100-mL round-bottom flask equipped with a reflux condenser. Approximately 100–150 mg of raw or thermally processed lipid samples have been refluxed for at least 1 h in the presence of 5 mL MeOH·BF_3_ solution in a water bath (maintained at a temperature with 5 °C higher than the boiling point of the mixture). When all lipid drops were dissolved, 10 mL of hexane was added to the mixture and the reflux process was continued for another 0.5 h. The hexane layer was separated in the top of the flask using sufficient saturated sodium chloride solution. The organic layer was collected in a gas chromatographic (GC) vial and dried over anhydrous sodium sulfate for 24 h in a cool and dark place. The hexane solution was then decanted and analyzed by gas chromatography-mass spectrometry (GC-MS).

### 2.3. Gas Chromatography—Mass Spectrometry Analysis of the Derivatized Lipid Fractions

Identification and quantification of FAMEs in the derivatized Mangalitza lipid fractions have been performed by GC-MS coupled technique. A GC-MS system comprised of a Hewlett Packard 6890 Series GC and a Hewlett Packard 5973 MS Detector (Agilent Technologies, Santa Clara, California, USA) was used. The GC conditions were: column Zebron 5-MS (30 m × 0.25 mm × 0.25 μm), column temperature starting from 50 °C for 1 min, 50–300 °C, with a heating rate of 6 °C/min and 300 °C for 5 min, solvent delay of 4 min, He flow of 1 mL/min, and sample volume of 1 μL. The MS conditions were: scanning range of 50–500 amu, ionization energy of 70 eV, and source temperature of 150 °C. Enhanced MSD ChemStation ver. D.02.00.275/2005 (Agilent Technologies, Santa Clara, California, USA) was used for acquisition and handling of the GC-MS data. The identification of the FAMEs in the derivatized Mangalitza lipid fractions was performed by comparing the experimental mass spectra (MS) with those from the NIST/EPA/NIH (National Institute of Standards and Technology/ Environmental Protection Agency/National Institutes of Health) Mass Spectral Library 2.0 (2011), NIST MS (National Institute of Standards and Technology Mass Spectrometry) Search 2.0 package (NIST, Gaithersburg, MD, USA), as well as by using the retention indices (RIs) obtained for the main FAMEs. The RI values were obtained by interpolating the retention times (RTs) by means of RI versus RT graph of the C_8_-C_20_ linear alkane standard mixture, analyzed in the same conditions. RIs of FAMEs from the derivatized Mangalitza lipid fractions were compared with the RIs of the standard FAMEs, analyzed in the same conditions.

### 2.4. Fourier-Transform Infrared Spectroscopy Analysis of the Lipid Fractions

Fourier-transform infrared spectroscopy analysis (FTIR) was performed in order to identify the presence of the specific groups in degraded lipid fractions (e.g., trans fats). A Bruker Vertex 70 FTIR (Bruker Optik GmbH, Ettlingen, Germany), equipped with a single-reflection Platinum diamond attenuated the total reflectance sampling module (ATR) and a deuterated lanthanum α-alanine doped triglycine sulphate detector (DLaTGS) has been used for FTIR analysis of the raw and thermally processed Mangalitza lipid fractions. The FTIR analysis conditions were as follows: wavenumber range of 4000–400 cm^−1^, sensitivity >0.5%, resolution of 4 cm^−1^, 128 scans 128, phase resolution of 32, sample mass of ~10–20 mg. The ATR-FTIR (attenuated the total reflectance—Fourier-transform infrared spectroscopy) data—OPUS ver. 7.2 software (Bruker Optik GmbH 2012, Ettlingen, Germany) was used for acquisition and handling of the FTIR data. The background spectra was acquired in the air for every sample analysis and the ATR was well cleaned using isopropanol (FTIR grade, Sigma-Aldrich, St. Louis, MO, USA) until no residual bands were observed in the background spectra.

### 2.5. Principal Component Analysis and Classical Statistical Analysis

Principal component analysis (PCA) is a powerful technique for discriminating between samples. PCA is widely used in food analysis, especially for physical chemical and sensory analyses or for evaluating the authenticity of food samples. PCA is generally used in order to extract the useful information from a large number of data. For food products, a raw matrix containing analysis results (e.g., rows containing sample types and columns containing independent variables such as in the case of GC-MS, where these variables are the relative concentrations of the main FAMEs and other compounds) is transformed in a product of two matrices by translation and rotation processes. First principal component (PC_1_ or Factor 1) is the direction in the properties space having a maximum covariance. The second principal component (PC_2_ or Factor 2) has the same property, but with the restriction of the orthogonality to the PC_1_. The next PCs can be obtained in a similar way. However, only a few PCs will be retained for evaluating the similarity/dissimilarity between the samples. The translation coordinates will provide the score plots, revealing the similarity/dissimilarity of the samples (cases), while the rotation polar coordinates will provide the loadings plots (variables), which indicate the influence of every variable for the classification of cases.

In the case of Mangalitza lipid fractions, GC-MS relative concentrations of all identified FAMEs have been considered as independent variables (columns) in the PCA analysis. Raw and thermally processed lipid fractions (duplicates) have been considered as cases in this GC-MS-PCA approach. The GC-MS-PCA analysis was performed using the PC and CA (Principal Components & Classification Analysis) module from Statistica 7.1 package (StatSoft). Centered data and a cross-validation method were used.

Classical statistical analysis of the data obtained from GC-MS analysis (relative concentrations) was performed by a one-way ANOVA (analysis of variance) module in Statistica 7.1 software. Results were presented both as mean ± standard deviation (SD) and as a data range.

## 3. Results and Discussion

### 3.1. Fatty Acid Profile of the Unprocessed and Thermally Processed Mangalitza Lipid Fractions

#### 3.1.1. Derivatization, Identification, and Quantification—General Considerations

**Figure 3:**
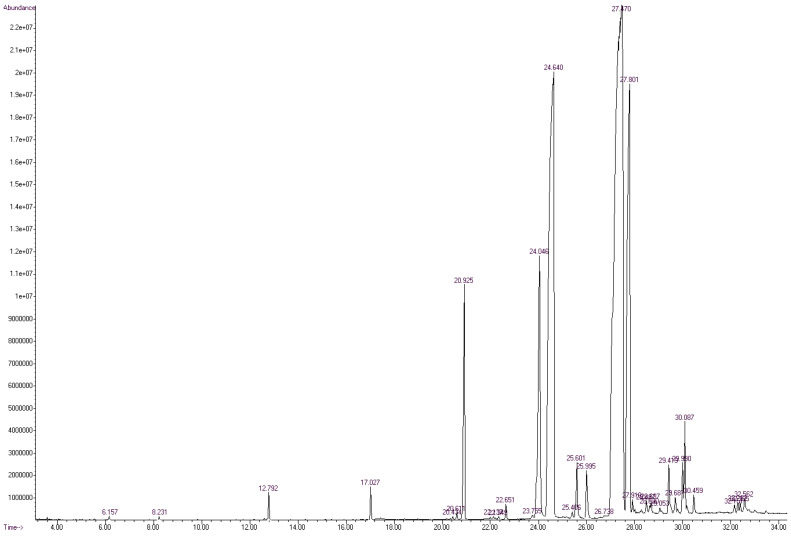
Gas chromatogram from the GC-MS analysis of the derivatized layer 1 of Mangalitza hard fat (raw sample, code U1). All other GC chromatograms for the raw samples are presented in the Supplementary material file (Figures S1, S3, S4, S7, and S8).

**Figure 4:**
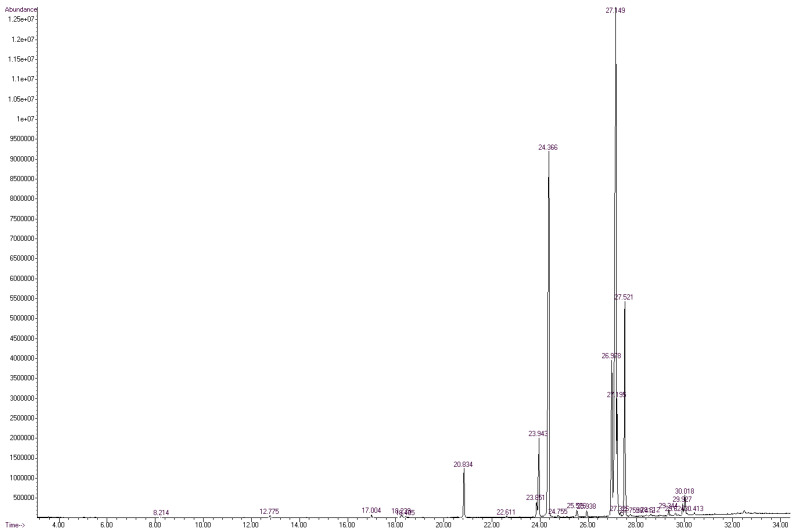
Gas chromatogram from the GC-MS analysis of the derivatized layer 1 of Mangalitza hard fat (thermally processed sample, code P1). All other GC chromatograms for the thermally processed samples are presented in the Supplementary material file (Figures S2, S5, S6, S9, and S10).

**Figure 5:**
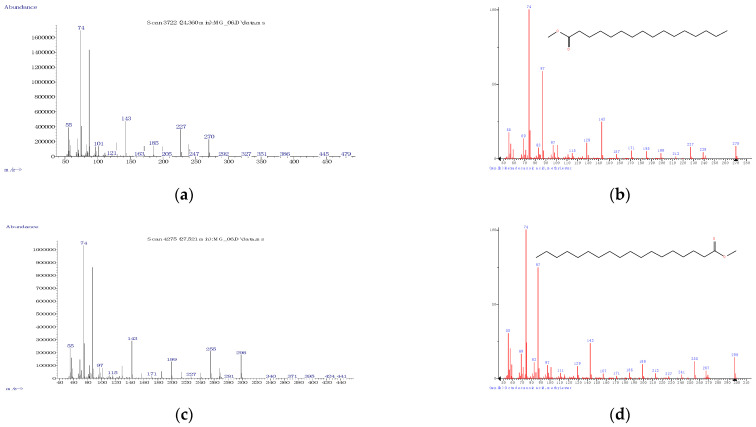
Experimental (left) and from the NIST 2011 database (right) mass spectra for the main fatty acids (as methyl esters) identified in the derivatized lipid fractions from Mangalitza hard fat—Saturated fatty acids (SFAs): palmitic acid (**a**,**b**) and stearic acid (**c**,**d**).

**Figure 6:**
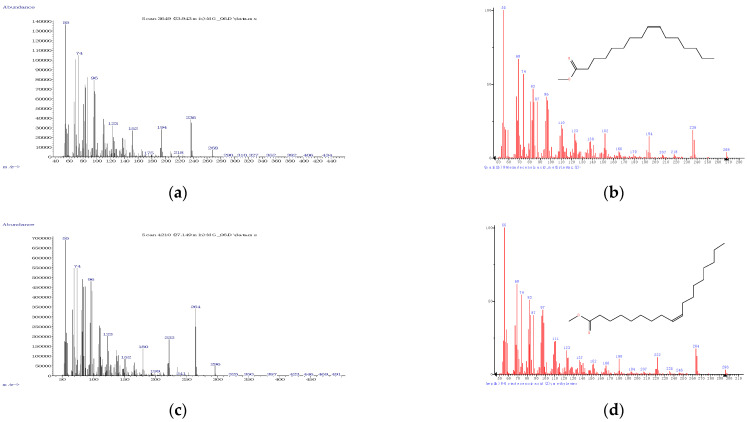

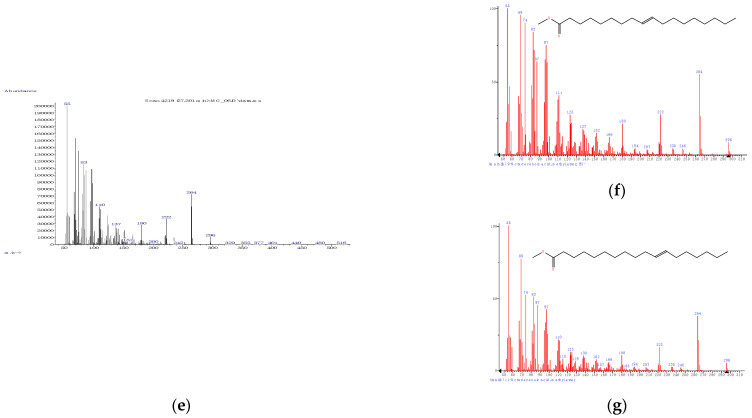
Experimental (left) and from the NIST 2011 database (right) mass spectra for the main fatty acids (as methyl esters) identified in the derivatized lipid fractions from Mangalitza hard fat—Monounsaturated fatty acids (MUFAs): palmitoleic acid (**a**,**b**), oleic acid (**c**,**d**), and vaccenic/elaidic acid (**e**–**g**).

**Figure 7:**
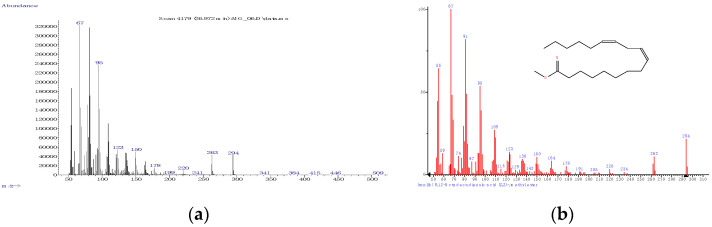
Experimental (left) and from the NIST 2011 database (right) mass spectra for the main fatty acids (as methyl esters) identified in the derivatized lipid fractions from Mangalitza hard fat—Polyunsaturated fatty acids (PUFAs): linoleic acid (**a**,**b**).

The FA profile of the specific layers of the hard fat of Mangalitza growing in the Northern Romania was evaluated by GC-MS analysis. The quantitative transesterification of the FA glycerides contained in the lipid fractions was performed by the BF_3_·methanol method. The corresponding FAMEs, as well as aldehyde dimethylacetals and dimethylacetals of the ω-formylated FAMEs that resulted from thermal degradation, have been expressed as relative concentrations (the percentage ratio between the GC area of a specific compound and the sum of areas of all separated compounds). The RTE integrator with a minimum of 10,000 area count and a baseline drop else tangent were used for automatic integration of the GC chromatograms. Up to 96 compounds were separated for the raw lipid fractions. However, only a few compounds have consistent relative concentrations in the region of 20–31 min (corresponding to RIs of 1686-2338, Figure 3 and Figure 4, as well as Figures S1–S10 for gas chromatograms, Figures S11a–v and S12a–h for the mass spectra in the Supplementary material file). All separated compounds have been identified with various matching probability by comparing experimental (actual) mass spectra (MS) with that from the NIST/EPA/NIH Mass Spectral Library 2.0 (2011) using two methods (Figure 5, Figure 6 and Figure 7): (1) PBM-Probability-Based Matching algorithm, developed at Cornell University by McLafferty and collaborators, respectively, the NIST MS Search algorithm [23,24,25], (2) calculation of the retention indices (RIs were obtained by interpolating the retention times in the RI versus RT graph for the C_8_-C_20_ linear alkanes analyzed under the same conditions), and comparing them with those obtained for the standard compounds (most of the standard FAMEs and degradation aldehydes were analyzed in previous studies) [26,27,28,29,30,31,32,33].

The main FAMEs have been identified with high probability by MS comparisons and their identities were confirmed by means of RIs. It is the case of palmitic acid, methyl ester (matching probability >81% for the first five hits in the NIST MS Search algorithm), stearic acid, methyl ester (matching probability >76% for the first four hits), and myristic acid, methyl ester (matching probability >82% for the first four hits) in the case of SFAs (Figure 5). In the case of MUFAs, the matching probability was lower, but the identities of compounds were confirmed by the second method based on RIs of the standard FAMEs. Thus, oleic acid methyl ester had the matching probability of only ~25%, but the identity was confirmed by the RI of ~2117 (Figure 6 and Table 1). Palmitoleic acid methyl ester had a matching probability >71% (for the first three hits) and RI of ~1917, while vaccenic and elaidic acids (as methyl esters, trans isomers) were not clearly separated by GC (they had matching probabilities lower than 14% and the RI of ~2120, Figure 6e–g and Table 1).

On the other hand, the most important PUFA was linoleic acid (methyl ester), with only ~37% probability of matching, but confirmed by the RI of the standard compound (~2103, Figure 7 and Table 1). MS matching probabilities differ by a few percent between sample types (e.g., raw and processed) and the previously mentioned probabilities are for the raw sample of Mangalitza hard fat, layer 1 (Figure 3). Other information on the GC-MS analysis of the raw and processed lipid layers of Mangalitza hard fat, as multiplicate determinations, can be found in the Supplementary material file (Figures S1–S10, S11a–v and S12a–h).

#### 3.1.2. Fatty Acid Profile of the Unprocessed Mangalitza Lipid Fractions

The most important fatty acids (as methyl esters) in the raw Mangalitza hard fat were MUFAs, especially oleic acid (Table 2, Table 3 and Table 4). It was identified in all fat layers at relative concentrations of 36.1–42.4%, and is less concentrated in layer 1. Palmitoleic acid (MUFA, RI 1917), was also identified at concentrations of 4.5–6.8%, which is slightly higher in layer 3 (near the skin). The presence of some trans fatty acids belonging to this class must be noticed. The main trans fatty acids were vaccenic and elaidic acids (as methyl esters). They can occur in the lipid layer or can be generated through isomerization reaction (elaidation in the case of elaidic acid) during the derivatization and especially the thermal and oxidative degradation (see below). Unfortunately, they were not separated by GC-MS under mentioned conditions and their cumulative concentration was in the range of 0.3–4.1% in the raw fat. Other MUFA identified in Mangalitza hard fat was the 11-eicosenoic acid, but at a lower concentration (0.9–1.5%). Generally, total MUFAs were a few percent higher in layers 2 and 3 of Mangalitza hard fat (48.59 ± 2.09% and 48.77 ± 1.01%) in comparison with those from layer 1 (45.05 ± 3.55%). The SFA class was less concentrated than the MUFA class with a total concentration ranging between 33.7–42.2% in the raw samples (Table 2, Table 3 and Table 4). Palmitic and stearic acids were the most concentrated, with values of 21.3–24.1% and 7.7–12.7%, respectively (Table 2, Table 3 and Table 4). Many other SFAs have been identified in the raw Mangalitza hard fat, but at lower concentrations. The presence of caprylic, capric, lauric, myristic, pentadecanoic, margaric, and arachidic acids were noted with concentrations lower than 1% except for myristic acid (2.6–3.3%). Moreover, a cyclic fatty acid, namely 2-hexylcyclopropaneoctanoic acid (methyl ester, RI 2012, identified with relatively low matching probability of ~38% for the first two hits) was identified in all samples at concentrations of 0.56–0.97%. Finally, PUFAs were less concentrated from all three fatty acid classes (7.1–16.2%, Table 2, Table 3 and Table 4). The major compound from this class was linoleic acid (5.8–14.9%), but other PUFAs were also identified at lower concentrations. They are arachidonic and 10,13-eicosadienoic acids, with concentrations in the raw fats of 0.4–0.7% and 0.7–1%, respectively. Fatty acids from the ω-3 class have been identified at low concentrations and not in all samples. Among these, (all-*Z*)-docosa-4,7,10,13,16,19-hexaenoic acid (DHA) was identified in some raw samples at a maximum of 0.12%, as methyl ester (RI 2402, data not presented).

Our results are in agreement with those from the literature. Straadt and co-workers compared the fatty acid profile of pork loins (intramuscular fat) from crossbreeds between Mangalitza and Duroc or Landrace/Yorkshire, which were fed ad libitum with conventional feed consisting of barley, wheat, and soy [9]. They found oleic acid as the main constituent, at a relative concentration of 42.2–42.8%, as methyl ester. The total MUFAs were in the range of 50.6–51.9%, which is slightly higher than our results on Mangalitza hard fat layers (42.5–50.1%, Table 2, Table 3 and Table 4). Among these, vaccenic acid was found at a relative concentration of 4.3–4.6%, but no information related to other trans fatty acids have been presented. On the other hand, SFAs and PUFAs were also similar to our results. Mangalitza crossbreeds had values of 38.8–39.7% and 8.1–8.5%, respectively [9]. A series of studies on the fatty acid profile of Mangalitza pigs have been performed by Parunović and collaborators [13,18,19,20,34]. They studied the effect of rearing, gender, or feeding on the fatty acid profile of different parts of Mangalitza varieties. Oleic acid was found at a relative concentration of 45.6% in the backfat of free-range reared Mangalitza and 49.6% for samples of the conventionally reared pigs [18]. They also identified the trans isomer elaidic acid at ~0.55% in these samples. On the other hand, no ω-3 long chain fatty acids have been identified (such as DHA). The fatty acid content was also determined in the *musculus longissimus thoracis et lumborum* from free-range and conventional reared Mangalitza pigs. The oleic acid content was 44.3–47% from a total of MUFA that ranged between 55.5–57.2%. The second important fatty acid was palmitic acid from the SFA class, which had a concentration of 23.2–24.6% in the corresponding lipid samples from free-range and conventional reared Mangalitza pigs [19]. Slightly higher oleic acid content (50.8%) has been obtained by the same research team in the case of lipid fraction of *musculus longissimus dorsi* from Swallow-belly Mangalitza reared in free-range conditions and fed with corn silage, feed flour, soybean, and sunflower oil meals, as well as minerals and vitamins [20,34].

The Mangalitza genotypes showed slight differences between the fatty acid profiles of the previously mentioned lipid fractions. MUFA contents are higher in the White Mangalitza genotype when compared with the Swallow-belly Mangalitza (~58% and 55.1%, respectively). On the contrary, an inverse order was observed for PUFA contents (5.2% and 7%, respectively) [13]. Another study compared the fatty acid profile of the lipid fractions of *longissimus dorsi* and *semitendinosus* of Mangalitza pigs reared in an extensive system and fed with an experimental diet based on flaxseed oil. They found an increase of the ω-3 α-linolenic acid of more than two times, compared with the control samples [16]. Similar study on the *musculus longissimus dorsi* fat composition of Mangalitza pigs that were fed with two types of feed based on sunflower seed and linseed revealed an oleic acid content of 42.3–43.9% and a total ω-3 PUFAs of 0.6–0.63% (including α-linolenic acid and DHA). On the other hand, ~4.5% of trans vaccenic acid, as well as ~0.13% conjugated linoleic acid have been identified [17]. However, our fatty acid profile data on Mangalitza “blonde” variety, reared in the Northern Romania in a free-range system and fed ad libitum with conventional feed, are close to those for other Mangalitza varieties reared and fed in the same conditions.

#### 3.1.3. Fatty Acid Profile and Degradation of the Thermally Processed Mangalitza Lipid Fractions

**Figure 8:**
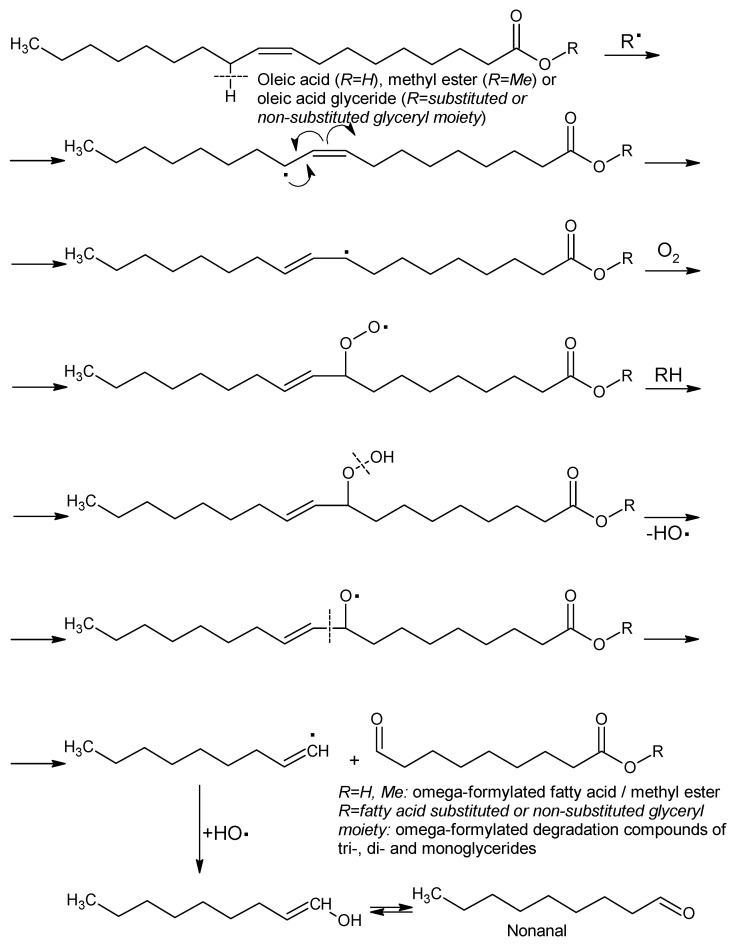
Proposed mechanism of thermal and oxidative degradation of a fatty acid glyceride or free fatty acid to the linear aldehydes and ω-formylated fatty acid derivatives (exemplification for oleic acid derivatives).

There are few studies related to the degradation of pork lipid fractions. Particularly, no such studies on Mangalitza fats have been found in the literature. However, it is difficult to quantify the degradation compounds of lipid components, especially if the degradation conditions are severe. It is the case of oxidative conditions at high temperatures, when polymers and volatile compounds can be generated. The first class of compounds is very difficult to characterize, while volatile compounds need specific analysis techniques for quantification due to partial loss by evaporation. In this study, the middle cooking conditions of Mangalitza hard fat were applied in order to evaluate the changes in the fatty acid profile, as well as the level of some degradation compounds from the linear aldehyde class. Mangalitza hard fat layers have been subjected to controlled heating at 130 °C for 30 min, under air and normal pressure. After cooling, the residue was treated with the derivatization reagent (BF_3_·MeOH followed by hexane dilution) at reflux until no insolubles remain. The free and bonded fatty acids (as mono-, di-, and triglycerides) are quantitatively derivatized to the corresponding FAMEs, while degradation aldehydes are derivatized to the corresponding dimethylacetals. Other degradation compounds such as ω-formylated fatty acids can be determined as dimethylacetals of the FAMEs [28,29,30,31,32,33]. However, the last compound class was identified at very low concentrations (<0.01%). By far, the most important degradation compounds in the thermally processed Mangalitza lipid layers were aldehydes, especially hexanal and nonanal (identified as dimethylacetals using appropriate standards, with RIs of 976 and 1280, respectively, Table 5). Hexanal concentration increased from 0.003% to 0.023–0.026% in the thermally processed lipid layers 2 and 3 (almost ten times higher), while nonanal had an increase of 4-5 times for these layers (from 0.005% to 0.019% for layer 2 and from 0.004% to 0.022% for layer 3). Malondialdehyde is also formed by oxidation/auto-oxidation and needs specific methods for detection and quantification (during the thermal and oxidative degradation, an important part of malondialdehyde was lost due to the lower boiling point of about 108 °C) [28,29,30,31,32,33,35]. Other higher aldehydes (saturated or unsaturated, e.g., C12:0 and C18:2 linear aldehydes) have also been identified in higher concentrations in the thermally and oxidative degraded lipid layers from Mangalitza hard fat, but with a lower matching probability (Table 5). The formation of linear aldehydes during the thermal and oxidative degradation starts from fatty acid glycerides or free fatty acids through a radical cleavage of a C-H bond from the vicinal position to a double bond. The unsaturated radical undergo a rearrangement to the more stable trans isomer radical, which can interact with an oxygen molecule to provide a peroxide radical. This will react with another fatty acid derivative (or other organic molecule) in order to provide a hydroperoxide derivative. Further radical cleavage at the peroxide bond will generate the oxi-radical that suffer another radical cleavage at the vicinal trans -C=C-C- σ bond. Two important intermediates have been formed, i.e., the ω-formylated fatty acid derivative (free, glyceride moiety or methyl ester for transesterified samples) and an unsaturated hydrocarbon radical. The interaction with the hydroxyl radical will generate an enol, which tautomerizes to the linear aldehyde as the final degradation compound (Figure 8) [28,29,30,31,32,33,35]. Hexanal can be generated in the same manner from ω-6 fatty acid glycerides or free acids. Unfortunately, only the remaining aldehydes from the degraded samples can be quantified by GC-MS because such volatile compounds will be partly evaporated during the lipid degradation.

There are small differences between the relative concentrations of the FAMEs corresponding to the thermally processed and raw lipid samples. No specific correlations with the concentrations of the main fatty acids have been observed. This is due to the physical chemical characteristics of compounds, mainly the volatility of the degradation aldehydes and susceptibility to polymerization of the unsaturated fatty acid glycerides. However, some specific compounds can be emphasized. It is the case of palmitoleic acid and trans MUFAs, i.e., vaccenic and elaidic acid. The relative concentration of methyl cis-palmitoleate decreases in all three thermally and oxidative degraded Mangalitza lipid layers (from 5.80% to 3.96% for layer 1, from 5.34% to 3.92% in layer 2, and from 6.68% to 5.27% in layer 3, Table 2, Table 3 and Table 4). On the contrary, trans-vaccenic and trans-elaidic acids (as methyl esters) appear at higher concentrations in the thermally processed lipid layers (from 2.21% to 4.21% for layer 1, from 2.25% to 4.54% in layer 2, and from 3.36% to 4.22% in layer 3, Table 2, Table 3 and Table 4). Generally, SFAs had almost no variation in the raw and degraded lipid layers (up to a difference of 1.5%), while these differences were in the range of 3.2–3.8% for both MUFA and PUFA concentrations in layers 2 and 3 (Table 2, Table 3 and Table 4).

### 3.2. Fourier-Transform Infrared Spectroscopy Analysis of the Unprocessed and Thermally Processed Mangalitza Lipid Fractions

**Figure 9:**
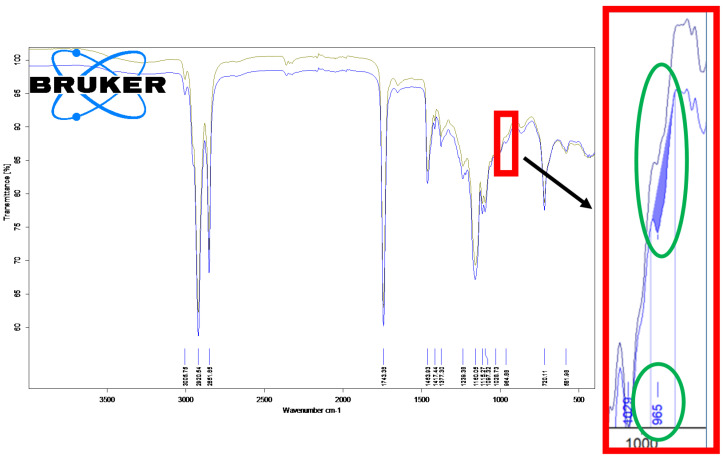
Superimposed Fourier-transform infrared spectroscopy (FTIR) spectra for the raw (unprocessed, green-U1) and thermally processed (blue-P1) Mangalitza lipid fraction (layer 1). The FTIR band at ~965 cm^−1^ is assigned for trans fatty acid glycerides and is more intense for the thermally processed sample (see the detail in red).

FTIR analysis allows us to identify the presence of specific groups in a mixture. The content of various compound classes can also be semi-quantitatively evaluated by this technique. There are two important FTIR regions that emphasize the characteristic absorption of various bonds in the raw and thermally degraded Mangalitza lipid fractions. The main compounds in these samples are triglycerides, but monoglycerides, diglycerides, free fatty acids, and aldehyde degradation compounds are also present. In the first region of about 3600–2700 cm^−1^, the band corresponding to OH and CH stretching appears. The second region of 1800–600 cm^-1^ is allocated to C=O and C-O stretching, C=C stretching, as well as bending and deformation of CH and C=C groups. There are no important differences between the FTIR band characteristics for the raw and degraded lipid fractions, except some variations on the intensities or wavenumbers for a few bonds. Thus, the =CH, as well as CH asymmetric and symmetric stretching appear in narrow ranges of 3006.2–3007.2 cm^−1^, 2952.7–2953.8 cm^−1^, 2917.2–2920.9 cm^−1^, and 2850.8–2852.1 cm^−1^, respectively (only the superimposed FTIR spectra for the raw and degraded Mangalitza lipid layer 1 is presented in Figure 9). One of the most important bands corresponding to ester C=O stretching of the triglycerides appear at 1738.6–1743.4 cm^−1^. Other weak or medium FTIR bands are those corresponding to CH deformation, CH_2_ bending, and CO stretching at 1461–1466 cm^−1^, ~1377/1239–1242/1159–1161, and 1114–1116/1097–1099/1060–1063/1028–1030 cm^−1^, respectively. It must be emphasized that the FTIR bands correspond to RCH=CHR’ groups. The C=C stretching appear in a wide range of 1650–1656 cm^−1^ for cis configuration. There is a clear difference between the raw and degraded lipid samples. The FTIR band appear at ~1650 cm^−1^ for the raw Mangalitza lipid fractions, while, for the thermally and oxidative degraded samples, this band appears at 1654–1656 cm^−1^. On the other hand, the rocking of C-H bending for cis RHC=CHR groups appear at 1417–1418 cm^−1^, without significant variation. Finally, the C=C bending for trans RHC=CHR groups appears at 963–966 cm^−1^, with slightly lower values for degraded samples (Figure 9). This band is more important for degraded samples with the band area being two-three times higher for degraded lipid layers 2 and 3, in comparison with the corresponding raw samples (0.016 and 0.030 absorbance unit·cm^−1^ for degraded samples and only 0.005–0.006 absorbance unit·cm^−1^ for the raw lipid layers 2 and 3, see Figure 9 for the integration technique for lipid layer 1 between 973–955 cm^−1^). There are many studies performing FTIR analysis of oils and fats, but less likely to evaluate the changes by degradation through FTIR. The main band corresponding to the isomerization of cis fatty acid derivatives to the harmful trans isomers can be clearly identified by FTIR at ~966 cm^−1^ [36]. This band was identified in turkey and pork ham slices after boiling, smoking, and roasting [37]. Other edible oils and fats have been classified by multivariate statistical techniques using FTIR data. It is the case of vegetable oils such as olive, sesame, hazelnut, canola, palm, soybean, cotton seed, rice bran, corn, coconut, or sunflower oils [38,39,40,41], fish oils [42,43], and animal fats, especially poultry lipids, lard, lamb, and cow fats [44,45,46]. The presence of cis-trans isomerization was evaluated in some edible oils [40,41,44].

### 3.3. Gas Chromatography-Mass Spectrometry—Principal Component Analysis (GC-MS-PCA) for the Raw and Thermally Processed Mangalitza Lipid Fractions

**Figure 10:**
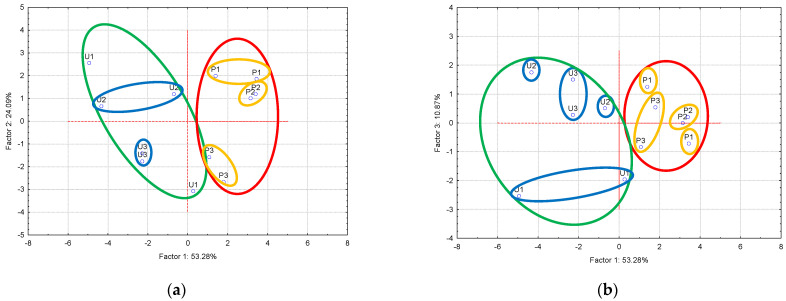

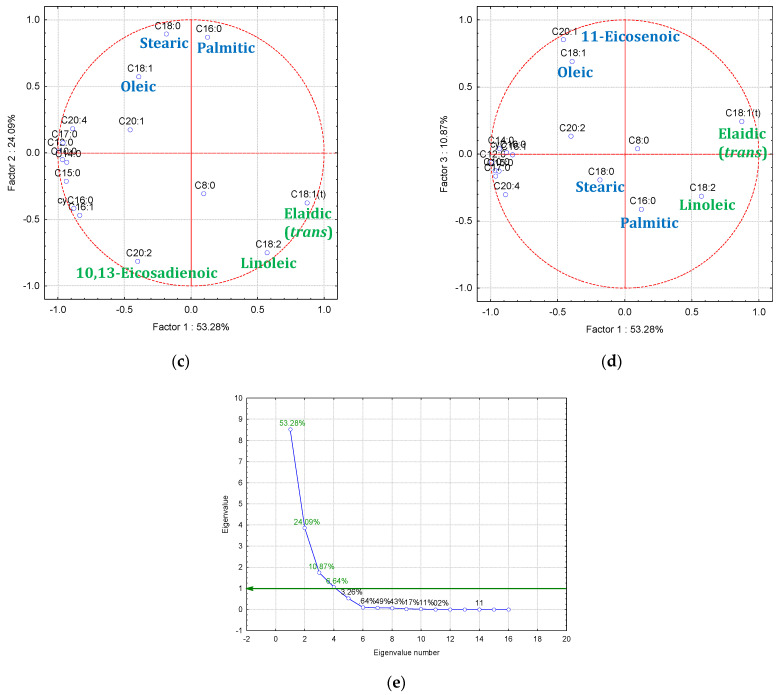
Principal Component Analysis (PCA) results for the gas chromatography-mass spectrometry (GC-MS) data of the derivatized lipid fractions from Mangalitza hard fat (relative concentrations data for specific fatty acid methyl esters, see Table 1 for codes. PC_1–3_ stand for Factors 1 to 3): (**a**) PC_2_ versus PC_1_ scores plot, (**b**) PC_3_ versus PC_1_ scores plot, (**c**) PC_2_ versus PC_1_ loadings plot, (**d**) PC_3_ versus PC_1_ loadings plot, (**e**) Eigenvalues of the correlation matrix (Principal Components, PCs, with significant influence to the explained variance are presented in green. Only a few PCs are sufficient to explain the variance of the data. In this case, the first four PCs explain 94.89% of the variance (instead of 16 raw variables). PCs with eigenvalues greater than 1 in the correlation matrix are considered to have a significant influence to the explained variance in the PCA technique). For other PCA results, see Supplementary material file (Tables S1–S6 and Figures S13–S16).

PCA is a valuable multivariate statistical analysis technique for evaluating the similarity-dissimilarity of samples (cases in PCA), i.e., raw Mangalitza lipid layers, as well as the corresponding thermally and oxidative degraded (processed) samples. It works with the raw data matrix (samples and variables) transformed by the translation and rotation in order to obtain a new coordinate named Principal Components (PCs) or Factors. First, this transformation is made in a way that provide maximum variance of the data for PC_1_. The second PC, PC_2_, is orthogonal on PC_1_, with the same observation related to the maximum variance (restricted by the orthogonality, and so on). Generally, only a few PCs are sufficient to explain the variance of the data (instead of the raw variables). On the other hand, the representation of translation coordinates gives the scores plot. Scores plot reveals the classification/grouping of cases (the similarity or dissimilarity of cases/samples). The representation of the cosines of the rotation angles gives the loadings plot. This reveals the influence of variables on the above-mentioned classification of the cases. Practically, PCA “transforms” variables into PCs (or Factors). Because it is difficult to compare all GC-MS data, taking into account the partial overlapping of information (e.g., the formation of aldehydes by oxidative degradation of unsaturated fatty acid glycerides generates an increase of the aldehyde content together with the decrease of the specific unsaturated fatty acid glycerides), the PCA technique helps the orthogonalization between variables. Sixteen variables (except those corresponding to C20:0, which was not identified in all samples, see Table 2, Table 3 and Table 4) representing the GC relative concentrations of all identified FAMEs in the raw and degraded Mangalitza lipid layers have been considered (variables —relative concentrations of the FAMEs—are coded in PCA as Cx:y, where x stands for carbon atoms and y for double bonds in cis configuration, except for t-trans configuration, cy-stands for fatty acids containing cyclopropane ring). Raw and processed lipid layers (samples/cases coded as U and P, respectively) were well discriminated by GC-MS-PCA analysis (Figure 10).

There is a clear discrimination between the raw (unprocessed, codes U) and thermally processed (codes P) Mangalitza lipid fractions along the first principal component, PC_1_, in the scores plot (Figure 10a,b). The explained variance for PC_1_ is 53.28%. Layer 1 samples were well classified in the upper side of the scores plot (U1 sample was an exception—outlier). Raw and processed samples corresponding to the lipid layers 2 and 3 are better grouped with each other in the middle and lower sides of the plot. The explained variance for PC_2_ was 24.09%, while, for PC_3_, it was 10.87%. However, the main four PCs were enough to retain the important information regarding the similarity-dissimilarity of the raw and processed Mangalitza lipid layers based on the GC-MS fatty acid profile (cumulative explained variance of 94.89%, Figure 10e, see the Supplementary material file for all detailed PCA results, Tables S1–S3). Loading plots provide information on the influence of variables for the classifications of cases. Relative concentrations of the linoleic and vaccenic/elaidic acids (as methyl esters) are the most important variables for discrimination between raw and processes lipid fractions (along PC_1_, Figure 10c,d). On the other hand, lipid layers are especially discriminated by palmitic, stearic, and oleic acids in the positive side of the PC_2_, as well as by linoleic and 10,13-eicosadienoic acids in the negative side (Figure 10c). The important variable for PC_3_ is the concentration of 11-eicosenoic acid (as a methyl ester, Figure 10d). It was observed that the GC-MS-PCA approach allows discrimination between raw and processed Mangalitza lipid fractions principally by relative concentrations of MUFAs, while between lipid layers by concentrations of SFAs (see Figure S13 and Tables S2 and S3 in the Supplementary material file). No significant differences were observed if the degradation aldehyde concentrations were introduced in the PCA, except the “localization” of the U and P groups. However, the PCA results based on both FAMEs and degradation aldehydes were also presented in the Supplementary material file (Figure S14). A similar comparison was performed for Mangalitza and other common pig (Danish Landrace variety grown in the same region, GC-MS data not published) hard fat samples. Mangalitza samples (both raw and processed) are completely dissimilar with the processed Landrace hard fat, especially based on the relative concentrations of linoleic and vaccenic/elaidic acids for PC_1_, oleic, and 11-eicosenoic acids for PC_2_. These mean that ω-6 PUFAs and trans ω-9 MUFAs are important for classification along PC_1_, while the same ω-9 MUFAs are also important for PC_2_. The explained variances for the first three PCs are 42.83%, 33.38%, and 18.66%, with a cumulative value of 94.88% (see Figures S15 and S16, Tables S4–S6 in the Supplementary material file).

There are many gas chromatography-principal component analysis (GC-PCA) coupled technique studies in the food field, including various edible oils and fats. Less studies were found for the application of GC-PCA discrimination in the case of pork lipids and no studies for Mangalitza lipid layers. PCA and orthogonal projections to latent structures—discriminant analysis (OPLS-DA) have been applied in order to discriminate between various oils and fats such as sunflower, canola, corn, soybean, rice bran, coconut, olive, palm oils, as well as lard, mutton, or beef tallows. Good classifications of these samples have been obtained based on GC-MS data. Moreover, the adulteration of canola oil by animal fat addition can be well established by partial least squares/projection to latent structures (PLS) [47]. Similar studies on many extra virgin olive oils have been performed. They were discriminated from tea, rapeseed, corn, sunflower, and sesame oils by PCA based on a fatty acid profile [48]. Some studies are related to the detection of adulteration of valuable oils by a GC-MS-PCA coupled technique. It is the case of flaxseed oil versus extra virgin olive oil for its geographical origin and variety [49,50,51]. Regarding the application of GC-MS-PCA on the discrimination of pork samples, only a few studies have been found. One of these deals with the classification of conventional, free range, and organic pork meat by means of ESI-MS/MS (electrospray ionization-tandem mass spectrometry) and PTR-MS (proton-transfer-reaction mass spectrometry), which were coupled with PCA. The best results were obtained for the discrimination of organic meat through a fatty acid profile [21]. On the other hand, the authentication of beef, pork, and chicken meat samples using triacylglycerol profiling have been performed using a DART-HRMS coupled technique (direct analysis in real time—high-resolution mass spectrometry) [22].

## 4. Conclusions

The fatty acid profile of the hard fat layers from Mangalitza variety reared in Northern Romania was evaluated. Both raw (unprocessed) and thermally and oxidative degraded (processed) samples have been assessed. MUFAs were the most concentrated in all Mangalitza hard fat layers, the concentration of oleic acid (as methyl ester) being in the range of 36.1–42.4%. Thermal and oxidative degradation of the lipid layers especially provide aldehydes and trans fatty acid derivatives, hexanal being identified at concentrations of eight times higher in the thermally processed layers near the skin. Similar increases were observed for nonanal, up to five times higher in the processed Mangalitza lipid layers. The formation of these aldehydes was exemplified through a radical mechanism of degradation of free fatty acids and their corresponding glycerides. The increase of the relative concentration of trans fatty acid glycerides was also demonstrated by both GC-MS and FTIR analyses. In order to extract the useful information from the large GC-MS data set, the coupling of this analysis with the multivariate statistical analysis, PCA, have been performed for the first time for Mangalitza lipid fractions. The unprocessed and thermally processed lipid samples were well discriminated by PUFAs and trans MUFAs, while the specific layers were discriminated by SFAs. The specific fatty acid profile of Mangalitza lipid layers was also discriminated from other landrace lipids. The GC-MS-PCA coupled technique can be useful for evaluating the type of pork lipids, as well as the level of degradation of various animal fats, but further studies are needed in order to calibrate such a technique for authenticity and quality evaluation of such animal products.

## Figures and Tables

**Table 1:** The codes of the main FAMEs identified by GC-MS in the derivatized Mangalitza lipid fractions. Retention time (RT) and retention index (RI) values have also been presented. RIs have been calculated on the basis of GC-MS data obtained for a C_8_-C_20_ linear alkane standard mixture.

Code ^1^	Fatty Acid, Methyl Ester (ω Class)	Class ^2^	Retention Time (RT) (Min)	Retention Index (RI)
C8:0	Caprylic acid, methyl ester	SFA	8.225 ± 0.012	1126.2 ± 0.5
C10:0	Capric acid, methyl ester	SFA	12.784 ± 0.012	1327.8 ± 0.6
C12:0	Lauric acid, methyl ester	SFA	17.018 ± 0.016	1528.5 ± 0.8
C14:0	Myristic acid, methyl ester	SFA	20.873 ± 0.032	1734.3 ± 1.8
C15:0	Pentadecanoic acid, methyl ester	SFA	22.636 ± 0.020	1835.9 ± 1.2
C16:1	Palmitoleic acid, methyl ester (ω-7)	MUFA	23.994 ± 0.039	1916.5 ± 2.3
C16:0	Palmitic acid, methyl ester	SFA	24.472 ± 0.081	1945.3 ± 4.9
cyC16:0	Cyclopropaneoctanoic acid, 2-hexyl-, methyl ester	cySFA	25.563 ± 0.028	2011.6 ± 1.7
C17:0	Margaric acid, methyl ester	SFA	25.969 ± 0.024	2036.4 ± 1.5
C18:2	Linoleic acid, methyl ester (ω-6)	PUFA	27.054 ± 0.051	2102.8 ± 3.2
C18:1	Oleic acid, methyl ester (ω-9)	MUFA	27.291 ± 0.099	2117.3 ± 6.0
C18:1(*t*)	Vaccenic/Elaidic acid, methyl ester (ω-11/9)	MUFA	27.328 ± 0.103	2119.6 ± 6.3
C18:0	Stearic acid, methyl ester	SFA	27.617 ± 0.083	2137.2 ± 5.0
C20:4	Arachidonic acid, methyl ester (ω-6)	PUFA	29.378 ± 0.028	2243.5 ± 1.7
C20:2	10,13-Eicosadienoic acid, methyl ester (ω-7)	PUFA	29.961 ± 0.028	2277.9 ± 1.7
C20:1	11-Eicosenoic acid, methyl ester (ω-9)	MUFA	30.053 ± 0.028	2283.3 ± 1.6
C20:0	Arachidic acid, methyl ester	SFA	30.434 ± 0.024	2305.5 ± 1.4

^1^ Cx:y stands for the FAME compound containing x carbon atoms and y double bonds in a cis configuration (except for t-trans configuration). ^2^ SFA-saturated fatty acid, MUFA-monounsaturated fatty acid, PUFA, polyunsaturated fatty acid, and cySFA-saturated fatty acid containing a cyclopropane ring.

**Table 2:** Relative concentrations of the main FAMEs identified by GC-MS analysis in the raw (unprocessed) and thermally processed lipid fraction (layer 1, codes U1 and P1) from Mangalitza hard fat. Values are expressed as mean ± SD and data range (in parenthesis).

Code	Relative Concentration, % (Layer 1, Unprocessed)	Relative Concentration, % (Layer 1, Processed)
C8:0	0.03 ± 0.01 (0.02–0.03)	0.04 ± 0.01 (0.03–0.04)
C10:0	0.21 ± 0.07 (0.16–0.26)	0.12 ± 0.01 (0.12–0.13)
C12:0	0.26 ± 0.07 (0.21–0.31)	0.15 ± 0.02 (0.14–0.17)
C14:0	2.96 ± 0.54 (2.58–3.34)	2.54 ± 0.20 (2.40–2.68)
C15:0	0.16 ± 0.02 (0.15–0.18)	0.06 ± 0.01 (0.06–0.07)
C16:1	5.80 ± 0.42 (5.5–6.10)	3.96 ± 0.21 (3.81–4.11)
C16:0	22.80 ± 1.90 (21.45–24.14)	23.53 ± 0.84 (22.94–24.13)
cyC16:0	0.84 ± 0.02 (0.83–0.86)	0.40 ± 0.06 (0.36–0.44)
C17:0	0.66 ± 0.19 (0.52–0.80)	0.32 ± 0.04 (0.29–0.35)
C18:2	10.38 ± 6.31 (5.92–14.85)	8.73 ± 1.85 (7.42–10.04)
C18:1	38.29 ± 3.07 (36.11–40.46)	40.62 ± 1.30 (39.71–41.54)
C18:1(*t*)	2.21 (0.27–4.14)	4.21 ± 0.12 (4.12–4.29)
C18:0	10.77 ± 2.96 (8.68–12.86)	10.84 ± 0.07 (10.79–10.89)
C20:4	0.56 ± 0.21 (0.41–0.71)	0.34 ± 0.09 (0.27–0.40)
C20:2	0.81 ± 0.21 (0.67–0.96)	0.61 ± 0.06 (0.57–0.65)
C20:1	0.96 ± 0.06 (0.92–1.00)	1.13 ± 0.22 (0.98–1.29)
C20:0	0.23 *	0.13 ± 0.04 (0.10–0.15)
ΣSFA	37.97 ± 5.93 (33.78–42.16)	37.74 ± 0.47 (37.41–38.07)
ΣMUFA	45.05 ± 3.55 (42.54–47.56)	45.72 ± 1.72 (44.50–46.94)
ΣPUFA	11.76 ± 6.31 (7.29–16.22)	9.68 ± 1.70 (8.48–10.88)

* one determination.

**Table 3:** Relative concentrations of the main FAMEs identified by GC-MS analysis in the raw (unprocessed) and thermally processed lipid fraction (layer 2, codes U2 and P2) from Mangalitza hard fat. Values are expressed as mean ± SD and data range (in parenthesis).

Code	Relative Concentration, % (Layer 2, Unprocessed)	Relative Concentration, % (Layer 2, Processed)
C8:0	0.03 ± 0.01 (0.02–0.03)	0.01 ± 0.00 (0.01–0.02)
C10:0	0.20 ± 0.03 (0.18–0.22)	0.09 ± 0.01 (0.08–0.10)
C12:0	0.26 ± 0.05 (0.22–0.30)	0.13 ± 0.02 (0.12–0.14)
C14:0	3.01 ± 0.45 (2.70–3.33)	2.35 ± 0.11 (2.27–2.43)
C15:0	0.15 ± 0.03 (0.13–0.17)	0.07 ± 0.00 (0.07–0.07)
C16:1	5.34 ± 1.20 (4.49–6.19)	3.92 ± 0.24 (3.75–4.10)
C16:0	22.12 ± 0.37 (21.86–22.38)	22.93 ± 0.19 (22.8–23.07)
cyC16:0	0.77 ± 0.29 (0.56–0.97)	0.44 ± 0.02 (0.43–0.45)
C17:0	0.63 ± 0.09 (0.56–0.69)	0.3 ± 0.00 (0.30–0.31)
C18:2	7.19 ± 1.98 (5.79–8.59)	10.73 ± 0.15 (10.63–10.84)
C18:1	41.88 ± 0.70 (41.39–42.38)	39.86 ± 0.08 (39.81–39.92)
C18:1(*t*)	2.25 ± 0.35 (2.00–2.49)	4.54 ± 0.15 (4.43–4.64)
C18:0	11.13 ± 0.34 (10.89–11.38)	10.15 ± 0.26 (9.97–10.33)
C20:4	0.45 ± 0.08 (0.39–0.50)	0.29 ± 0.01 (0.28–0.30)
C20:2	0.81 ± 0.01 (0.80–0.82)	0.63 ± 0.04 (0.60–0.66)
C20:1	1.36 ± 0.19 (1.23–1.49)	1.08 ± 0.05 (1.04–1.12)
C20:0	0.18 ± 0.02 (0.17–0.19)	0.12 ± 0.00 (0.12–0.12)
ΣSFA	37.71 ± 0.04 (37.68–37.73)	36.17 ± 0.30 (35.95–36.38)
ΣMUFA	48.59 ± 2.09 (47.11–50.07)	44.87 ± 0.11 (44.79–44.94)
ΣPUFA	8.45 ± 1.89 (7.11–9.79)	11.65 ± 0.21 (11.50–11.79)

**Table 4:** Relative concentrations of the main FAMEs identified by GC-MS analysis in the raw (unprocessed) and thermally processed lipid fraction (layer 3, codes U3 and P3) from Mangalitza hard fat. Values are expressed as mean ± SD and data range (in parenthesis).

Code	Relative Concentration, % (Layer 3, Unprocessed)	Relative Concentration, % (Layer 3, Processed)
C8:0	0.02 ± 0.00 (0.02–0.03)	0.06 ± 0.03 (0.04–0.09)
C10:0	0.20 ± 0.00 (0.19–0.20)	0.15 ± 0.01 (0.15–0.16)
C12:0	0.25 ± 0.01 (0.24–0.26)	0.17 ± 0.02 (0.15–0.18)
C14:0	3.13 ± 0.11 (3.05–3.21)	2.79 ± 0.25 (2.62–2.97)
C15:0	0.16 ± 0.01 (0.15–0.17)	0.09 ± 0.01 (0.08–0.09)
C16:1	6.68 ± 0.23 (6.52–6.84)	5.27 ± 0.40 (4.99–5.56)
C16:0	21.53 ± 0.32 (21.31–21.76)	21.38 ± 0.96 (20.70–22.06)
cyC16:0	0.94 ± 0.01 (0.94–0.95)	0.61 ± 0.04 (0.58–0.64)
C17:0	0.54 ± 0.01 (0.53–0.54)	0.36 ± 0.01 (0.35–0.36)
C18:2	9.77 ± 1.20 (8.92–10.62)	13.06 ± 0.13 (12.97–13.15)
C18:1	40.85 ± 1.16 (40.03–41.67)	38.67 ± 0.80 (38.1–39.23)
C18:1(*t*)	3.36 ± 0.17 (3.24–3.48)	4.22 ± 0.78 (3.67–4.77)
C18:0	7.88 ± 0.20 (7.73–8.02)	8.14 ± 0.23 (7.97–8.31)
C20:4	0.42 ± 0.01 (0.41–0.43)	0.35 ± 0.02 (0.34–0.37)
C20:2	0.85 ± 0.04 (0.82–0.88)	0.82 ± 0.12 (0.74–0.90)
C20:1	1.24 ± 0.07 (1.19–1.29)	1.03 ± 0.04 (1.00–1.06)
C20:0	0.17 ± 0.05 (0.13–0.20)	0.10 ± 0.01 (0.09–0.11)
ΣSFA	33.87 ± 0.29 (33.67–34.07)	33.24 ± 0.96 (32.56–33.92)
ΣMUFA	48.77 ± 1.01 (48.06–49.49)	44.98 ± 0.44 (44.66–45.29)
ΣPUFA	11.04 ± 1.15 (10.23–11.85)	14.24 ± 0.03 (14.22–14.26)

**Table 5:** Relative concentrations (%) of some degradation compounds (mainly aldehydes, as dimethylacetals) identified by GC-MS analysis in the lipid fraction from Mangalitza hard fat (unprocessed lipid layers—U1-U3 and thermally processed lipid layers—P1-P3). Ald-Cx:y stands for an aldehyde with x carbon atoms and y double bonds, while AldAc-Cx:y stands for an ω-formylated fatty acid with the same characteristics. Values are expressed as mean ± SD and data range (in parenthesis).

Sample Code	Ald-C6:0	Ald-C9:0	AldAc-C12:0 ^1^	Ald-C18:2 ^1^
**Retention index (RI)**	976.0 ± 0.5	1280.0 ± 0.6	1600.7 ± 0.8	1703.1 ± 2.9
**Retention time (RT)** (min)	5.037 ± 0.011	11.714 ± 0.013	18.420 ± 0.015	20.314 ± 0.052
U1	0.013 ± 0.012 (0.005–0.022)	0.009 ± 0.005 (0.005–0.012)	0.018 ± 0.005 (0.014–0.021)	0.008 ± 0.004 (0.006–0.011)
P1	0.018 ± 0.001 (0.017–0.019)	0.022 ± 0.002 (0.020–0.023)	0.060 ± 0.023 (0.043–0.076)	0.015 ± 0.008 (0.010–0.021)
U2	0.003 ± 0.001 (0.002–0.003)	0.005 ± 0.001 (0.004–0.005)	0.009 ± 0.001 (0.008–0.009)	0.009 ± 0.002 (0.007–0.010)
P2	0.023 ± 0.007 (0.018–0.027)	0.019 ± 0.002 (0.018–0.020)	0.042 ± 0.002 (0.040–0.043)	0.011 ± 0.004 (0.008–0.014)
U3	0.003 ± 0.000 (0.002–0.003)	0.004 ± 0.001 (0.003–0.004)	0.010 ± 0.003 (0.008–0.012)	0.010 ± 0.003 (0.007–0.012)
P3	0.026 ± 0.006 (0.022–0.030)	0.022 ± 0.009 (0.016–0.028)	0.032 ± 0.002 (0.030–0.033)	0.007 ± 0.003 (0.005–0.009)

^1^ Identified by mass spectra (MS) comparison, with low matching probability.

## Data Availability

The data presented in this study are available in the Supplementary material file.

## Supplementary Materials

The following are available online at https://www.mdpi.com/2304-8158/10/2/242/s1. Figure S1: Gas chromatogram from the GC-MS analysis of the derivatized layer 1 of Mangalitza hard fat (raw sample, code U1, duplicate “b”-top, superimposed GC chromatograms for duplicates “a” and “b”-bottom), Figure S2: Gas chromatogram from the GC-MS analysis of the derivatized layer 1 of Mangalitza hard fat (thermally processed sample, code P1, duplicate “b”-top, superimposed GC chromatograms for duplicates “a” and “b”-bottom). Figure S3: Gas chromatogram from the GC-MS analysis of the derivatized layer 2 of Mangalitza hard fat (raw sample, code U2, duplicate “a”). Figure S4: Gas chromatogram from the GC-MS analysis of the derivatized layer 2 of Mangalitza hard fat (raw sample, code U2, duplicate “b”-top, superimposed GC chromatograms for duplicates “a” and “b”-bottom). Figure S5: Gas chromatogram from the GC-MS analysis of the derivatized layer 2 of Mangalitza hard fat (thermally processed sample, code P2, duplicate “a”). Figure S6: Gas chromatogram from the GC-MS analysis of the derivatized layer 2 of Mangalitza hard fat (thermally processed sample, code P2, duplicate “b”-top. Superimposed GC chromatograms for duplicates “a” and “b”-bottom). Figure S7: Gas chromatogram from the GC-MS analysis of the derivatized layer 3 of Mangalitza hard fat (raw sample, code U3, duplicate “a”). Figure S8: Gas chromatogram from the GC-MS analysis of the derivatized layer 3 of Mangalitza hard fat (raw sample, code U3, duplicate “b”-top, superimposed GC chromatograms for duplicates “a” and “b”-bottom). Figure S9: Gas chromatogram from the GC-MS analysis of the derivatized layer 3 of Mangalitza hard fat (thermally processed sample, code P3, duplicate “a”). Figure S10: Gas chromatogram from the GC-MS analysis of the derivatized layer 3 of Mangalitza hard fat (thermally processed sample, code P3, duplicate “b”-top, superimposed GC chromatograms for duplicates “a” and “b”-bottom). Figure S11: Experimental (left) and from the NIST 2011 database (right) mass spectra for the fatty acids (as methyl esters) identified in the derivatized lipid fractions from Mangalitza hard fat: caprylic acid (a and b), capric acid (c and d), lauric acid (e and f), myristic acid (g and h), pentadecanoic acid (i and j), 2-hexylcyclopropaneoctanoic acid (k and l), margaric acid (m and n), arachidonic acid (o and p), 10,13-eicosadienoic acid (q and r), 11-eicosenoic acid (s and t), and arachidic acid (u and v). Figure S12: Experimental (left) and from the NIST 2011 database (right) mass spectra for the main degradation aldehydes (as dimethylacetals) identified in the derivatized lipid fractions from thermally processed Mangalitza hard fat: hexanal (a and b), nonanal (c and d), ω-formylated fatty acid-C12:0 (as dimethylacetal and methyl ester, e and f), and aldehyde-C18:2 (g and h). Table S1: Factor coordinates of cases, based on correlations, for the PCA results for the GC-MS data of the derivatized lipid fractions from Mangalitza hard fat (U and P for unprocessed and processed samples, relative concentrations data for specific fatty acid methyl esters, or relative concentrations data for fatty acid methyl esters classes—SFA: saturated FAs, MUFA: monounsaturated FAs and PUFA: polyunsaturated FAs). Table S2: Factor coordinates of the variables, based on correlations, for the PCA results for the GC-MS data of the derivatized lipid fractions from Mangalitza hard fat (relative concentrations data for specific fatty acid methyl esters, or relative concentrations data for fatty acid methyl esters classes— SFA: saturated FAs, MUFA: monounsaturated FAs, and PUFA: polyunsaturated FAs). Table S3: Eigenvalues of correlation matrix, and related statistics, for the PCA results for the GC-MS data of the derivatized lipid fractions from Mangalitza hard fat (relative concentrations data for specific fatty acid methyl esters, or relative concentrations data for fatty acid methyl esters classes—SFA: saturated FAs, MUFA: monounsaturated FAs and PUFA: polyunsaturated FAs). Figure S13: PCA results for the GC-MS data of the derivatized lipid fractions from Mangalitza hard fat (relative concentrations data for fatty acid methyl esters class—SFA: saturated FAs. MUFA: monounsaturated FAs and PUFA: polyunsaturated FAs, o-6/7/9-ω class, t-stands for trans): (**a**) PC_2_ versus PC_1_ scores plot. (**b**) PC_3_ versus PC_1_ scores plot. (**c**) PC_2_ versus PC_1_ loadings plot. (**d**) PC_3_ versus PC_1_ loadings plot. (**e**) Eigenvalues of the correlation matrix (PCs with a significant influence for the explained variance are presented in green). Figure S14: PCA results for the GC-MS data of the derivatized lipid fractions from Mangalitza hard fat (relative concentrations data for specific fatty acid methyl esters and for degradation aldehyde dimethylacetals): (**a**) PC_2_ versus PC_1_ scores plot. (**b**) PC_3_ versus PC_1_ scores plot. (**c**) PC_2_ versus PC_1_ loadings plot. (**d**) PC_3_ versus PC_1_ loadings plot. (**e**) Eigenvalues of the correlation matrix (PCs with a significant influence for the explained variance are presented in green). Table S4: Factor coordinates of cases, based on correlations, for the PCA results for the GC-MS data of the derivatized lipid fractions from Mangalitza hard fat—Mg_U1/2/3 and Mg_P1/2/3 (or U and P for unprocessed and processed samples) in comparison with landrace pig hard fat—Pk/P_Pk (relative concentrations data for specific fatty acid methyl esters, or relative concentrations data for fatty acid methyl esters class—SFA: saturated FAs, MUFA: monounsaturated FAs, and PUFA: polyunsaturated FAs). Table S5: Factor coordinates of the variables, based on correlations, for the PCA results for the GC-MS data of the derivatized lipid fractions from Mangalitza hard fat—Mg_U1/2/3 and Mg_P1/2/3 (or U and P for unprocessed and processed samples) in comparison with landrace pig hard fat—Pk/P_Pk (relative concentrations data for specific fatty acid methyl esters, or relative concentrations data for fatty acid methyl esters class—SFA: saturated FAs, MUFA: monounsaturated FAs, and PUFA: polyunsaturated FAs). Table S6: Eigenvalues of correlation matrix, and related statistics, for the PCA results for the GC-MS data of the derivatized lipid fractions from Mangalitza hard fat—Mg_U1/2/3 and Mg_P1/2/3 (or U and P for unprocessed and processed samples) in comparison with landrace pig hard fat—Pk/P_Pk (relative concentrations data for specific fatty acid methyl esters or relative concentrations data for fatty acid methyl esters class—SFA: saturated FAs, MUFA: monounsaturated FAs, and PUFA: polyunsaturated FAs). Figure S15: PCA results for the GC-MS data of the derivatized lipid fractions from Mangalitza hard fat—Mg_U1/2/3 and Mg_P1/2/3 in comparison with landrace pig hard fat—Pk (relative concentrations data for specific fatty acid methyl esters): (**a**) PC_2_ versus PC_1_ scores plot. (**b**) PC_3_ versus PC_1_ scores plot. (**c**) PC_2_ versus PC_1_ loadings plot. (**d**) PC_3_ versus PC_1_ loadings plot. (**e**) Eigenvalues of the correlation matrix (PCs with a significant influence for the explained variance are presented in green). Figure S16: PCA results for the GC-MS data of the derivatized lipid fractions from Mangalitza hard fat—U and P, in comparison with landrace pig hard fat—P_Pk (relative concentrations data for fatty acid methyl esters class—SFA: saturated FAs, MUFA: monounsaturated FAs, and PUFA: polyunsaturated FAs. o-6/7/9 -ω class, t-stands for trans): (**a**) PC_2_ versus PC_1_ scores plot, (**b**) PC_3_ versus PC_1_ scores plot, (**c**) PC_2_ versus PC_1_ loadings plot, (**d**) PC_3_ versus PC_1_ loadings plot, and (**e**) Eigenvalues of the correlation matrix (PCs with a significant influence on the explained variance are presented in green).
