# Supplementary material for: Fatty Acid Profile of Lipid Fractions of Mangalitza (Sus scrofa domesticus) from Northern Romania: A GC-MS-PCA Approach
Source: Foods. 2021 Jan 26;10(2):242. doi: 10.3390/foods10020242 (PMC7912583; doi:10.3390/foods10020242)
Supplement: Supplementary file 1 [file foods-10-00242-s001.pdf]

*foods*

*Article*

# Supplementary material

# Fatty acid profile of lipid fractions of Mangalitza (*Sus scrofa domesticus*) from Northern Romania: a GC-MS – PCA approach

**Cornelia Petroman [1,†], Gabriela Popescu [2,†], Raymond-Nandy Szakal [3], Virgil Păunescu [4,5], Lavinia P. Drăghia [4,5], Gabriel S. Bujancă [6], Cosmina A. Chirilă [3], Daniel I. Hădărugă [7], Loredana Văduva [2], Nicoleta G. Hădărugă [3,*], Ioan Petroman [2]**

1 Department II – Economy and Company Financing, Banat's University of Agricultural Sciences and Veterinary Medicine "King Michael I of Romania" from Timişoara, Calea Aradului 119, 300645 –Timişoara, Romania; corneliapetroman@usab-tm.ro (C.P.)

2 Department of Rural Management and Development, Banat's University of Agricultural Sciences and Veterinary Medicine "King Michael I of Romania" from Timişoara, Calea Aradului 119, 300645 –Timişoara, Romania; gabrielapopescu@usab-tm.ro (G.P.); loredanavaduva@usab-tm.ro (L.V.); ioan_petroman@usab-tm.ro (I.P.)

3 Department of Food Science, Banat's University of Agricultural Sciences and Veterinary Medicine "King Michael I of Romania" from Timişoara, Calea Aradului 119, 300645 –Timişoara, Romania; raymondnalbu@gmail.com (R.N.S.); cosminachirila@yahoo.com (C.A.C.); nicoletahadaruga@usab-tm.ro; nico_hadaruga@yahoo.com (N.G.H.)

4 Department of Physiology and Immunology, "Victor Babeş" University of Medicine and Pharmacy, Eftimie Murgu Sq. 2, 300041 – Timişoara, Romania; vpaunescu@umft.ro (V.P.); draghia_lavinia@yahoo.com (L.P.D.)

5 Centre for Gene and Cellular Therapies in the Treatment of Cancer – OncoGen, Clinical County Hospital of Timişoara, Liviu Rebreanu Blvd. 156, 300736 – Timişoara, Romania

6 Department of Food Control, Banat's University of Agricultural Sciences and Veterinary Medicine "King Michael I of Romania" from Timişoara, Calea Aradului 119, 300645 –Timişoara, Romania; gabrielbujanca@yahoo.com (G.S.B.)

7 Department of Applied Chemistry, Organic and Natural Compounds Engineering, Polytechnic University of Timişoara, Carol Telbisz 6, 300001 – Timişoara, Romania; daniel.hadaruga@upt.ro (D.I.H.)

* Correspondence: nico_hadaruga@yahoo.com; Tel.: +40-256-277-423 (N.G.H.)

† These authors contributed equally to this work. They are both principal authors.

Received: 27 December 2020; Accepted: 21 January 2021; Published: 26 January 2021

## 1. Gas chromatography – mass spectrometry analysis of the derivatized lipid fractions

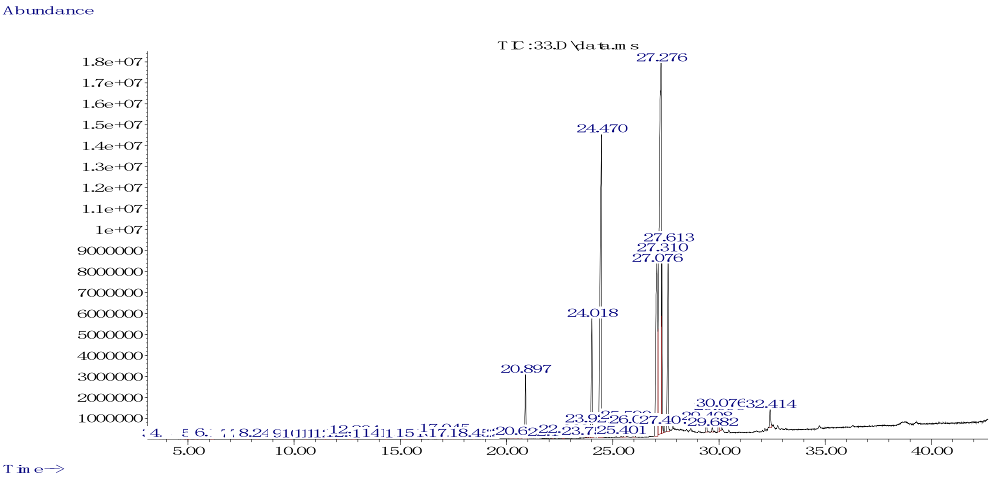


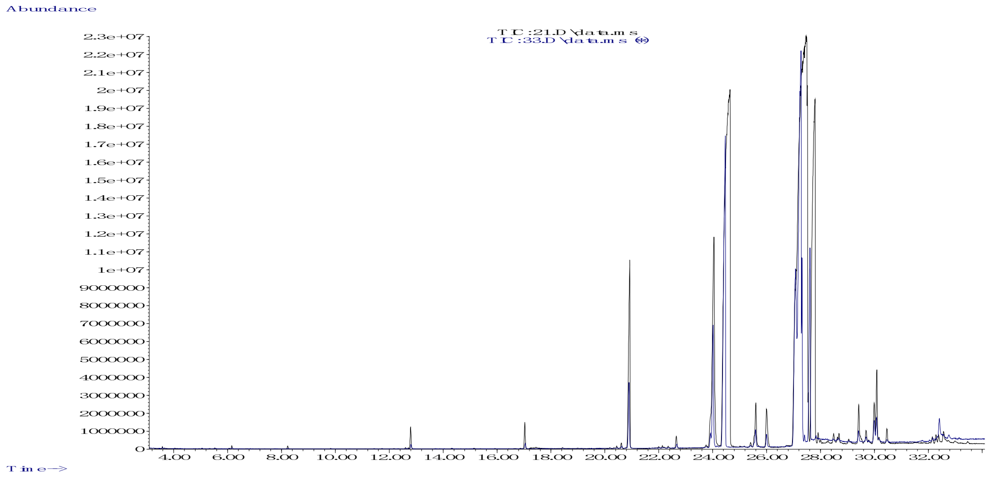


**Figure S1.** Gas chromatogram from the GC-MS analysis of the derivatized layer 1 of Mangalitza hard fat (raw sample, code U1, duplicate “b” – top; superimposed GC chromatograms for duplicates “a” and “b” – bottom).

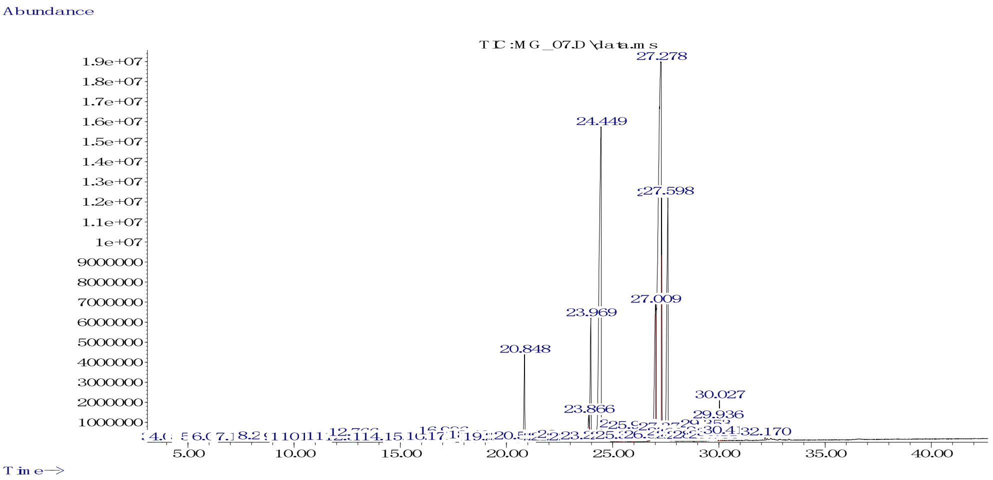

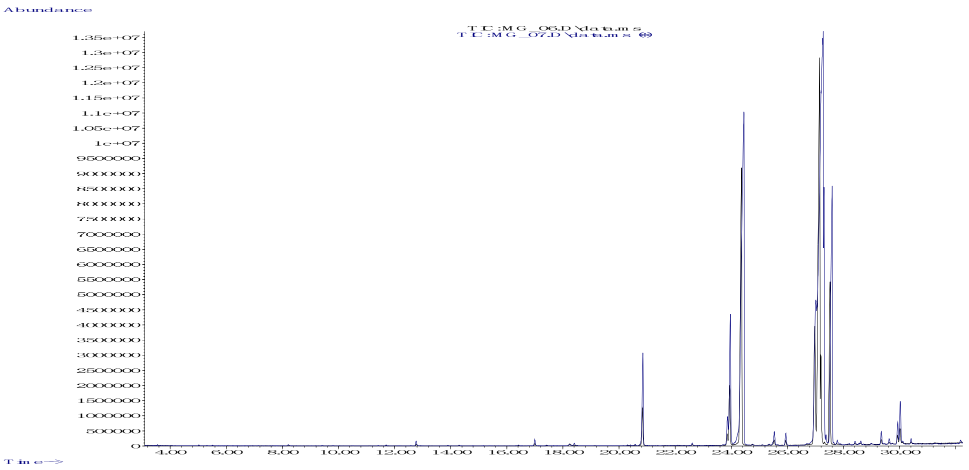

**Figure S2.** Gas chromatogram from the GC-MS analysis of the derivatized layer 1 of Mangalitza hard fat (thermally processed sample, code P1, duplicate "b" – top; superimposed GC chromatograms for duplicates "a" and "b" – bottom).

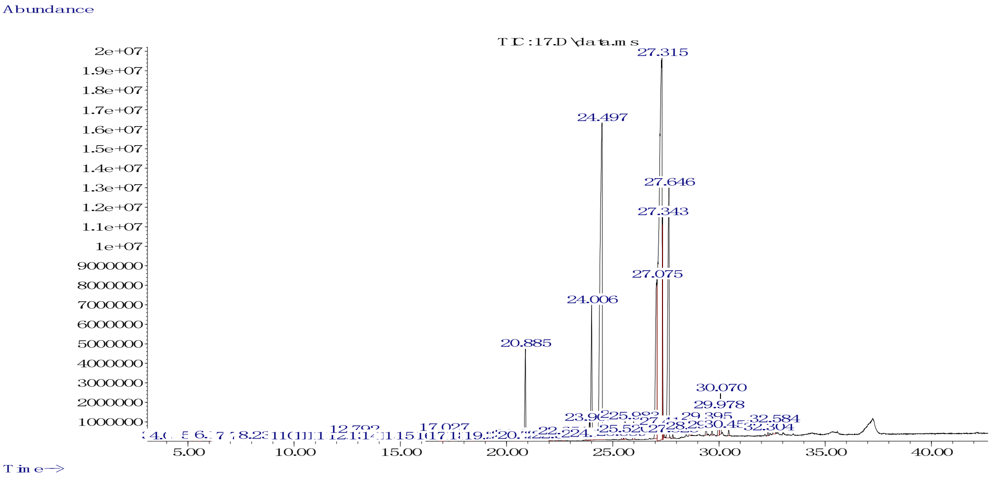


**Figure S3.** Gas chromatogram from the GC-MS analysis of the derivatized layer 2 of Mangalitza hard fat (raw sample, code U2, duplicate "a").

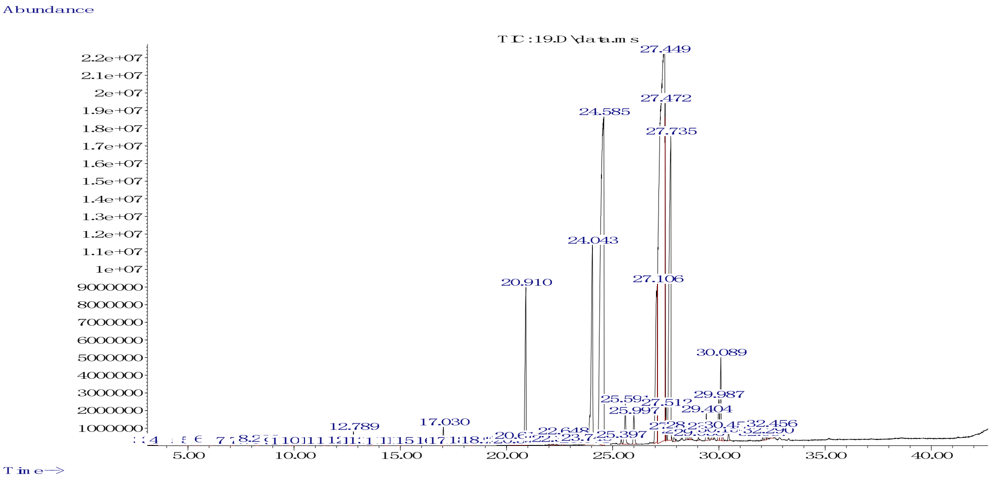

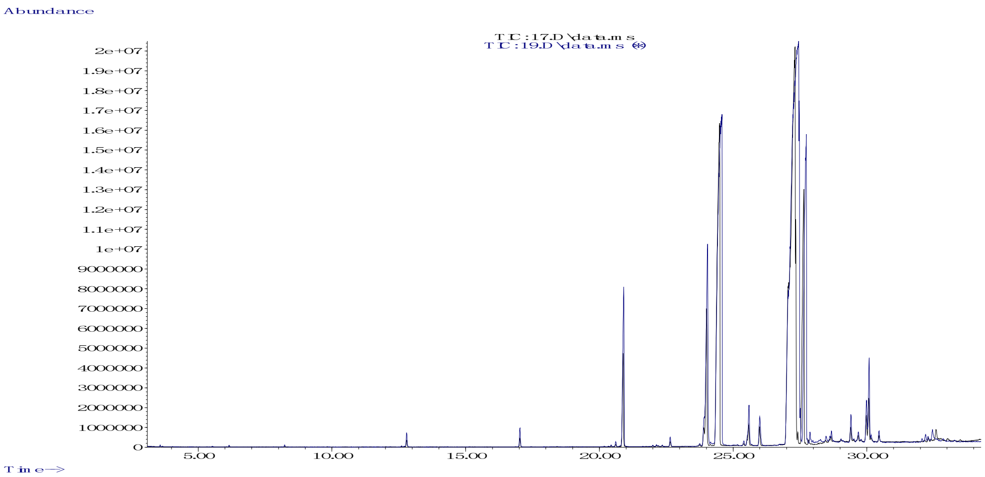


**Figure S4.** Gas chromatogram from the GC-MS analysis of the derivatized layer 2 of Mangalitza hard fat (raw sample, code U2, duplicate "b" – top; superimposed GC chromatograms for duplicates "a" and "b" – bottom).

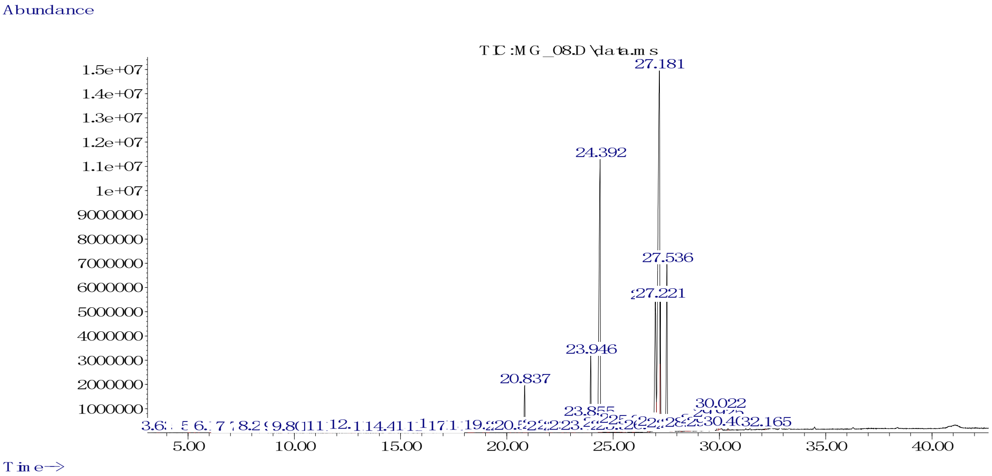


**Figure S5.** Gas chromatogram from the GC-MS analysis of the derivatized layer 2 of Mangalitza hard fat (thermally processed sample, code P2, duplicate "a").

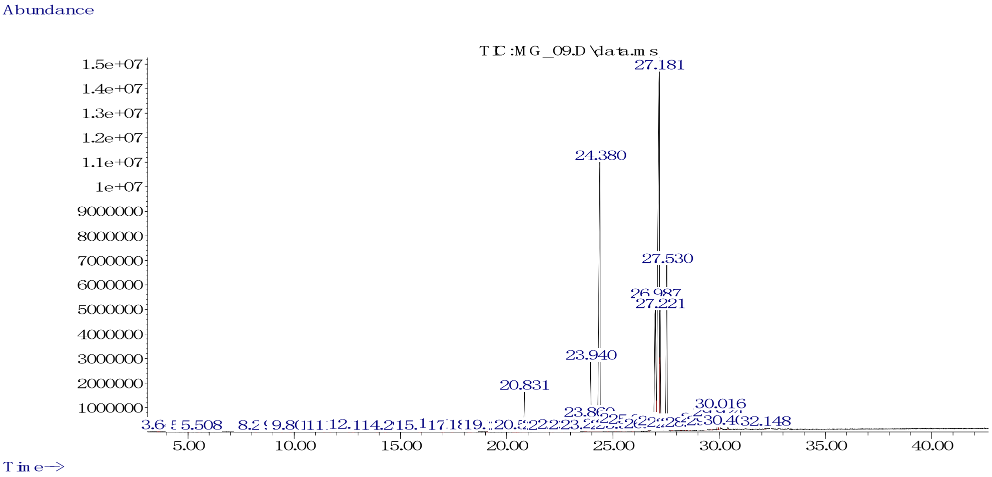

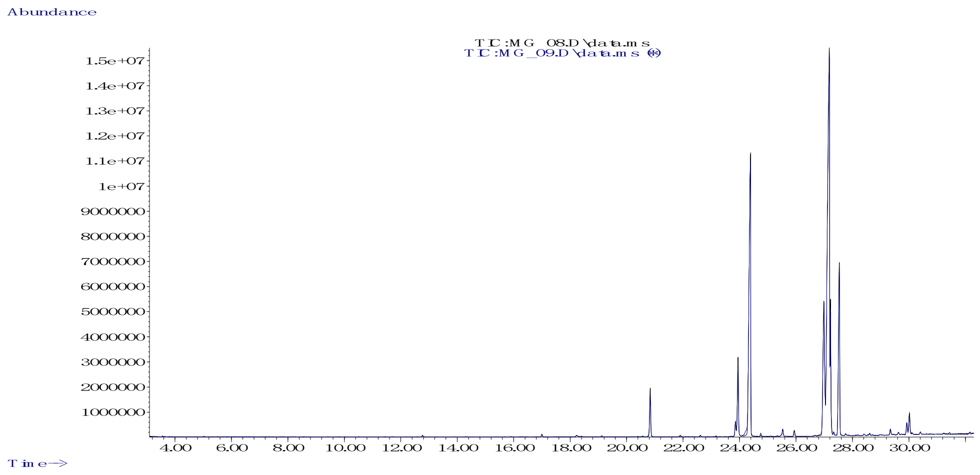


**Figure S6.** Gas chromatogram from the GC-MS analysis of the derivatized layer 2 of Mangalitza hard fat (thermally processed sample, code P2, duplicate "b" – top; superimposed GC chromatograms for duplicates "a" and "b" – bottom).

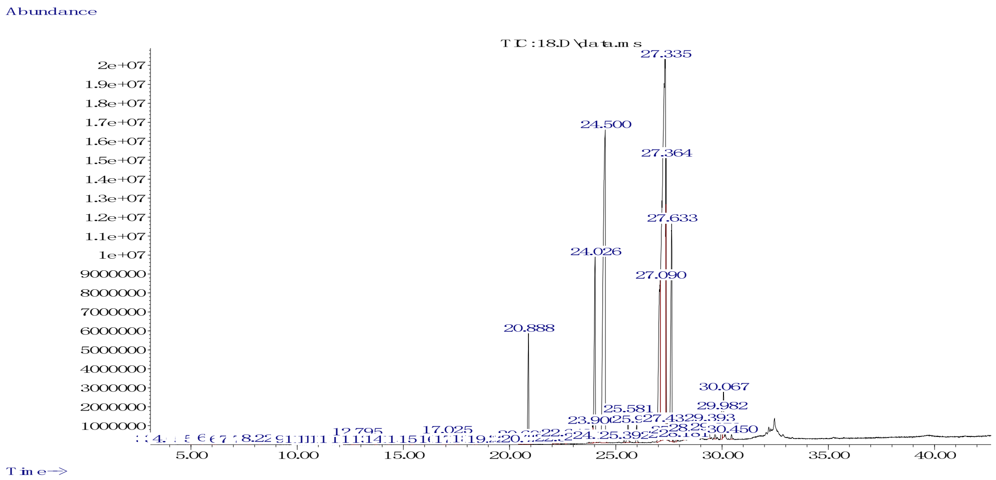


**Figure S7.** Gas chromatogram from the GC-MS analysis of the derivatized layer 3 of Mangalitza hard fat (raw sample, code U3, duplicate "a").

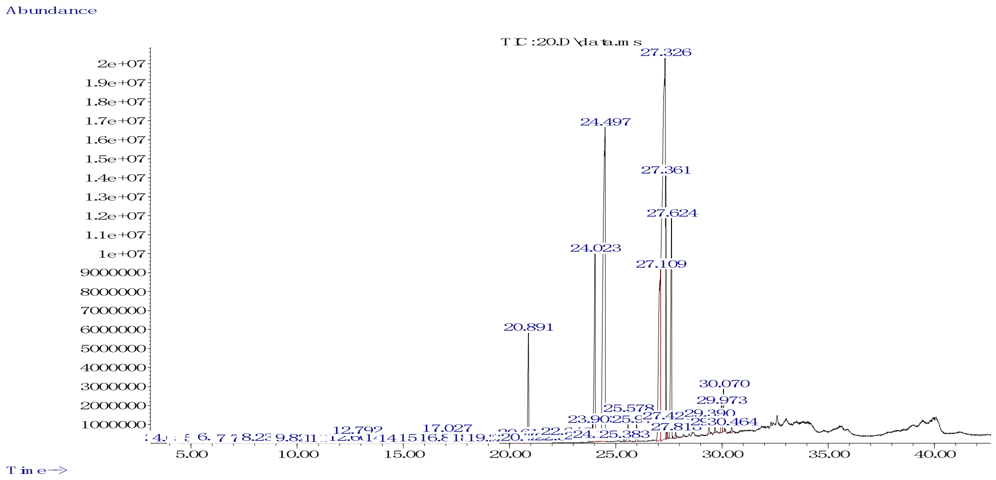

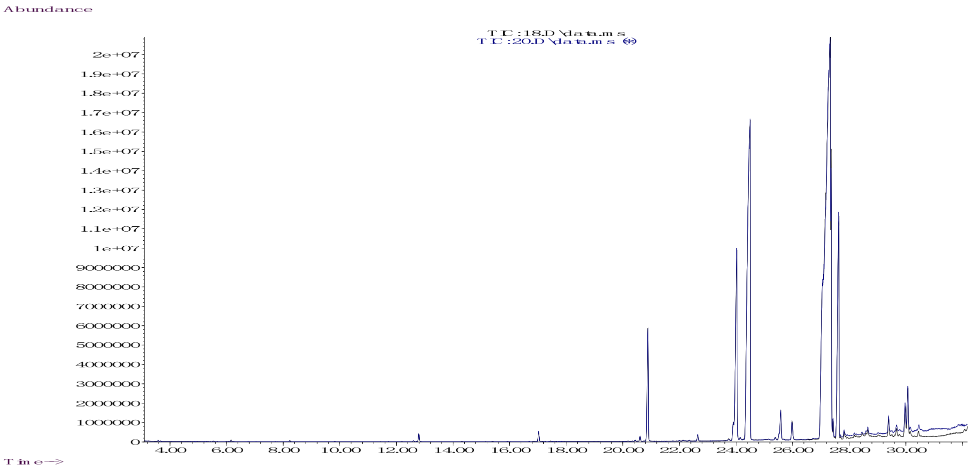


**Figure S8.** Gas chromatogram from the GC-MS analysis of the derivatized layer 3 of Mangalitza hard fat (raw sample, code U3, duplicate "b" – top; superimposed GC chromatograms for duplicates "a" and "b" – bottom).

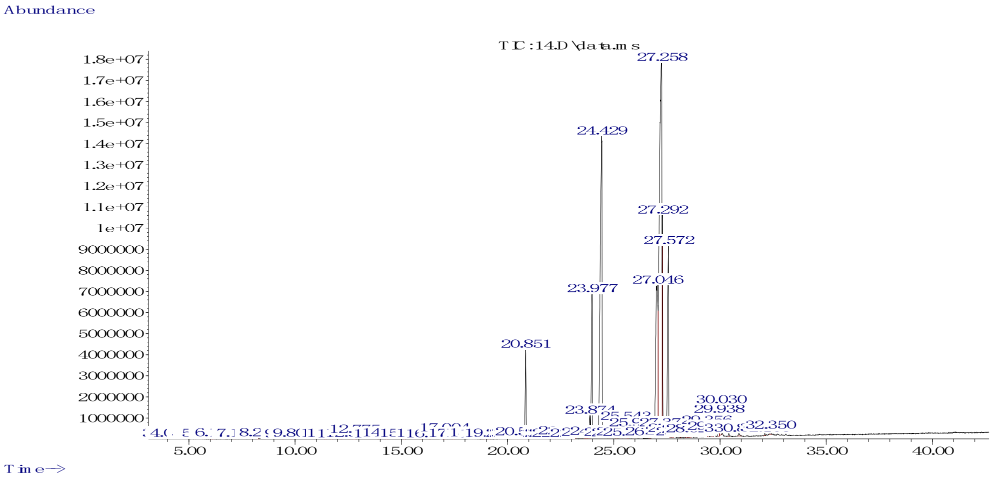


**Figure S9.** Gas chromatogram from the GC-MS analysis of the derivatized layer 3 of Mangalitza hard fat (thermally processed sample, code P3, duplicate "a").

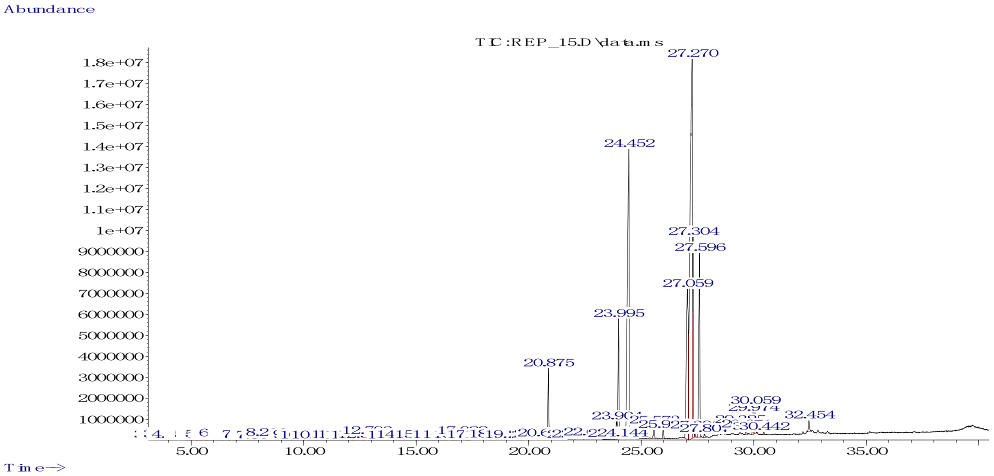

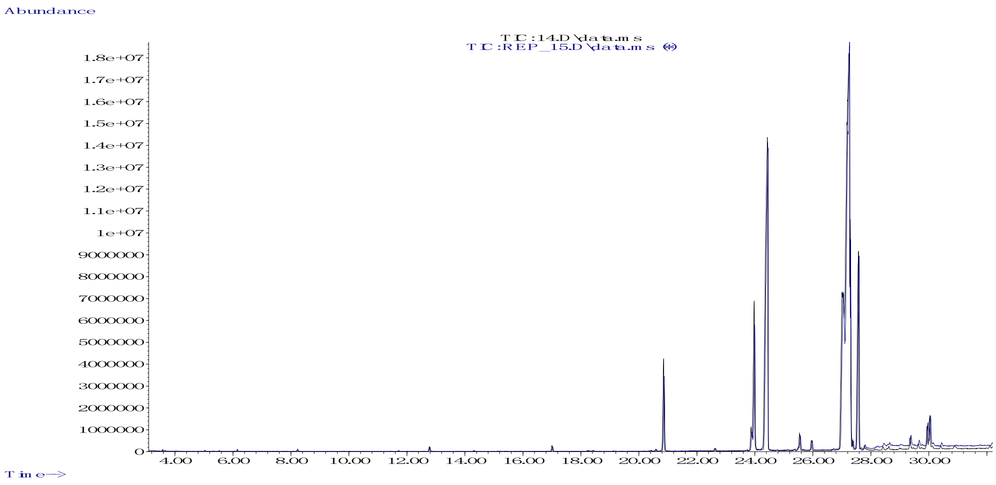


**Figure S10.** Gas chromatogram from the GC-MS analysis of the derivatized layer 3 of Mangalitza hard fat (thermally processed sample, code P3, duplicate "b" – top; superimposed GC chromatograms for duplicates "a" and "b" – bottom).

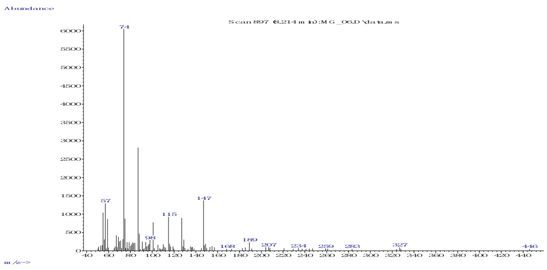

(**a**)

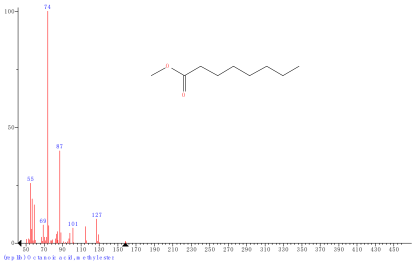

(**b**)

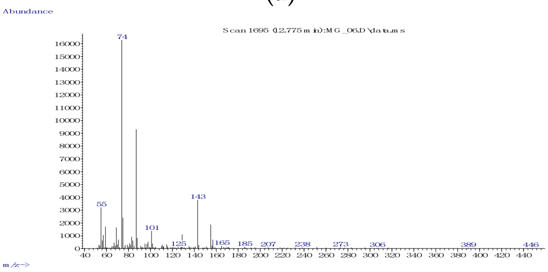

(**c**)

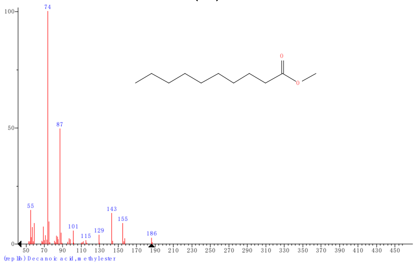

(**d**)

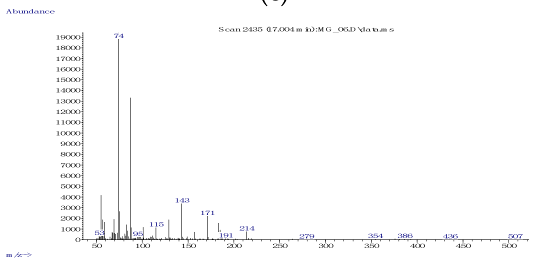

(**e**)

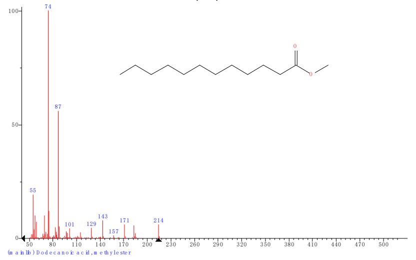

(**f**)

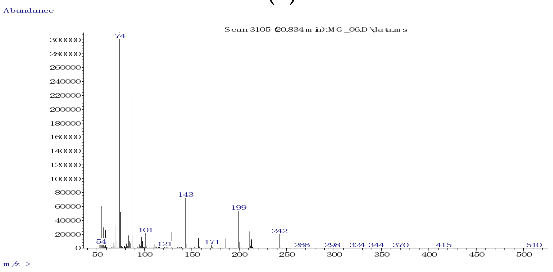

(**g**)

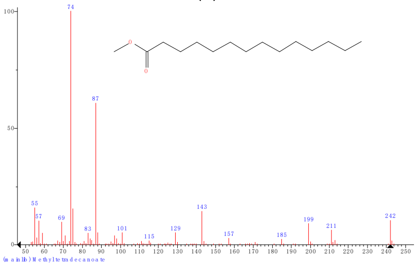

(**h**)

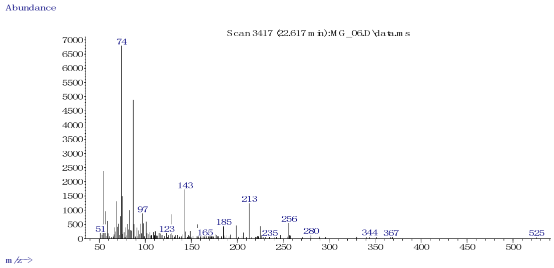


**(i)**

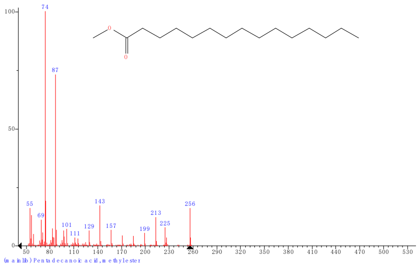


**(j)**

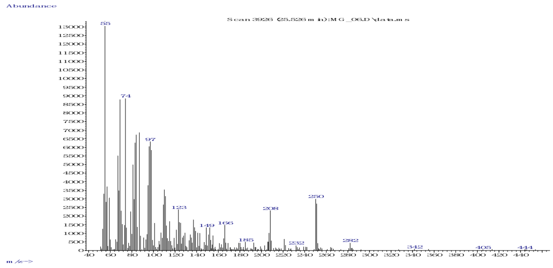


**(k)**

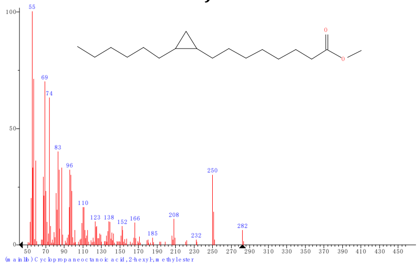


**(l)**

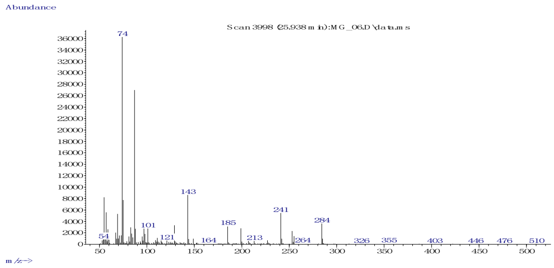


**(m)**

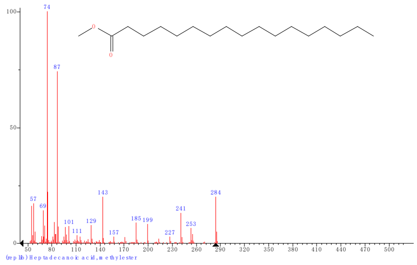


**(n)**

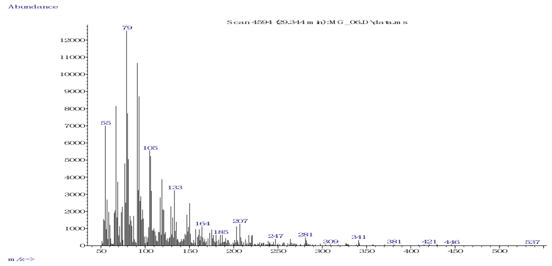


**(o)**

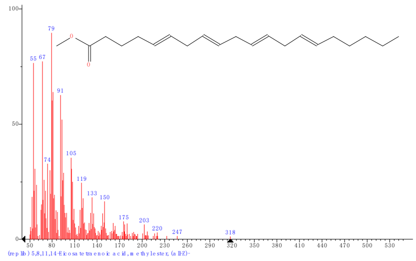


**(p)**

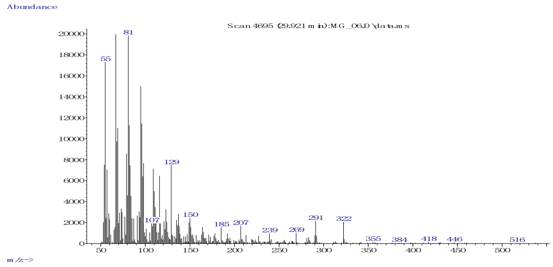


**(q)**

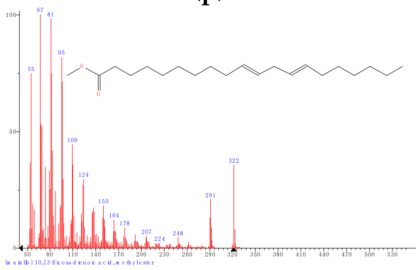


**(r)**

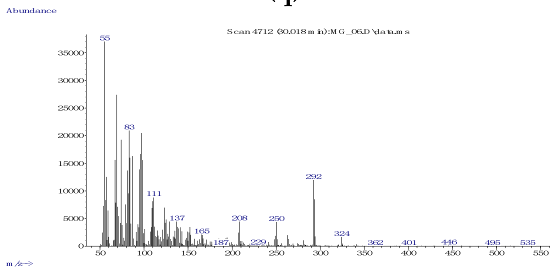


**(s)**

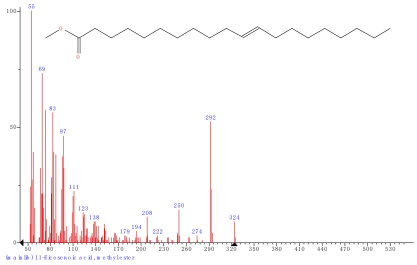


**(t)**

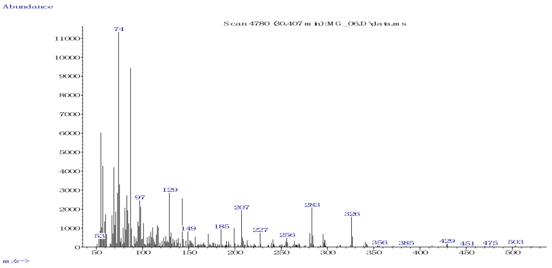


**(u)**

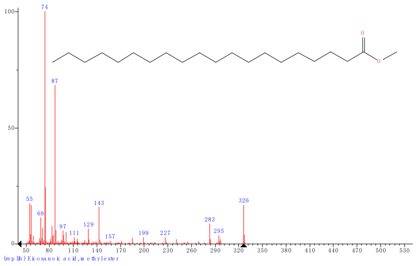

**(v)**

**Figure S11.** Experimental (left) and from the NIST 2011 database (right) mass spectra for the fatty acids (as methyl esters) identified in the derivatized lipid fractions from Mangalitza hard fat: caprylic acid (a and b), capric acid (c and d), lauric acid (e and f), myristic acid (g and h), pentadecanoic acid (i and j), 2-hexylcyclopropaneoctanoic acid (k and l), margaric acid (m and n), arachidonic acid (o and p), 10,13-eicosadienoic acid (q and r), 11-eicosenoic acid (s and t), and arachidic acid (u and v); MS spectra for the main fatty acid (as mehyl esters) presented in the main text are excepted.

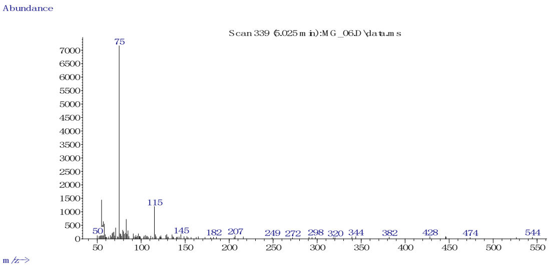


(**a**)

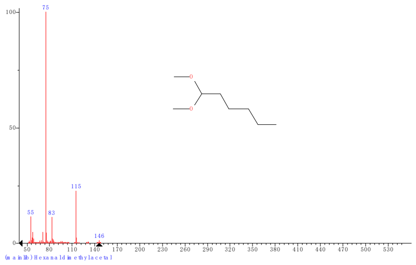

(**b**)

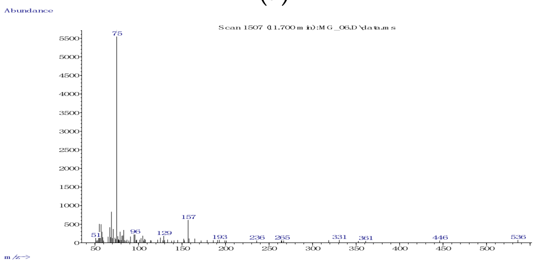


(**c**)

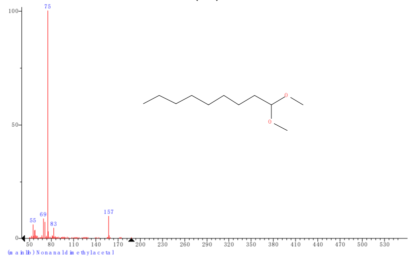

(**d**)

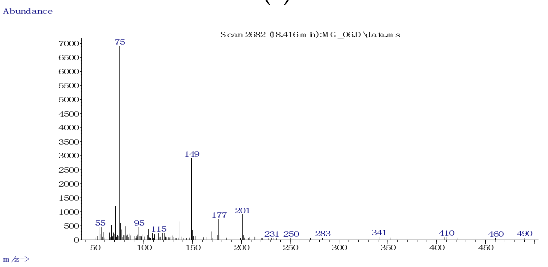


(**e**)

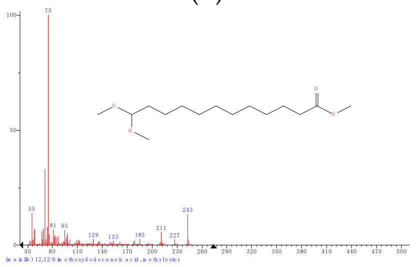

(**f**)

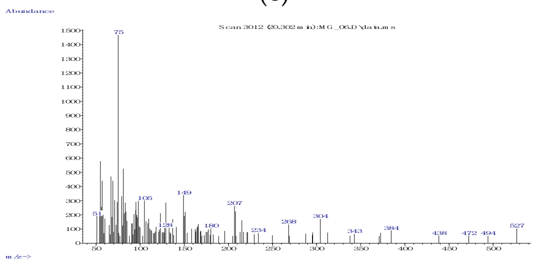


(**g**)

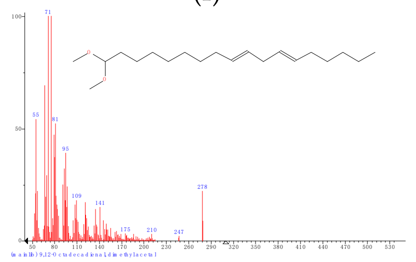

(**h**)

**Figure S12.** Experimental (left) and from the NIST 2011 database (right) mass spectra for the main degradation aldehydes (as dimethylacetals) identified in the derivatized lipid fractions from thermally processed Mangalitza hard fat: hexanal (a and b), nonanal (c and d), ω-formylated fatty acid-C12:0 (as dimethylacetal and methyl ester, e and f), and aldehyde-C18:2 (g and h).

## 2. Principal component analysis of the GC-MS data of the derivatized lipid fractions

**Table S1.** Factor coordinates of cases, based on correlations, for the PCA results for the GC-MS data of the derivatized lipid fractions from Mangalitza hard fat (U and P for unprocessed and processed samples; relative concentrations data for specific fatty acid methyl esters, or relative concentrations data for fatty acid methyl esters classes – SFA: saturated FAs, MUFA: monounsaturated FAs and PUFA: polyunsaturated FAs).

| Case | Fact. 1 | Fact. 2 | Fact. 3 | Fact. 4 | Fact. 5 | Fact. 6 | Fact. 7 | Fact. 8 | Fact. 9 | Fact. 10 | Fact. 11 |
|---|---|---|---|---|---|---|---|---|---|---|---|
| **U1** | -4.943 | 2.557 | -2.537 | 0.781 | 0.167 | -0.075 | 0.155 | -0.196 | -0.027 | 0.056 | -0.016 |
| **U1** | 0.280 | -3.076 | -1.966 | -0.934 | -1.135 | -0.131 | 0.209 | 0.240 | -0.003 | -0.080 | 0.025 |
| **U2** | -0.689 | 1.199 | 0.506 | -0.158 | -1.281 | 0.314 | -0.513 | -0.289 | 0.063 | -0.107 | 0.003 |
| **U2** | -4.336 | 0.660 | 1.758 | 0.145 | -0.262 | -0.142 | -0.147 | 0.536 | -0.108 | 0.113 | 0.017 |
| **U3** | -2.276 | -1.406 | 1.507 | -0.561 | 0.322 | 0.230 | 0.373 | -0.344 | 0.074 | 0.061 | 0.095 |
| **U3** | -2.278 | -1.754 | 0.279 | -0.969 | 0.964 | 0.281 | -0.039 | 0.050 | 0.041 | -0.119 | -0.119 |
| **P1** | 3.452 | 1.858 | -0.711 | 0.645 | 0.519 | 0.580 | 0.076 | 0.369 | 0.094 | -0.058 | 0.049 |
| **P1** | 1.393 | 1.995 | 1.259 | 0.294 | 0.026 | -0.568 | 0.260 | -0.024 | 0.091 | -0.251 | -0.009 |
| **P2** | 3.136 | 1.008 | -0.010 | -0.993 | 0.267 | -0.002 | -0.010 | -0.145 | -0.442 | 0.015 | 0.004 |
| **P2** | 3.405 | 1.200 | 0.206 | -0.893 | -0.366 | -0.122 | 0.096 | -0.031 | 0.203 | 0.279 | -0.064 |
| **P3** | 1.068 | -1.569 | -0.839 | 0.071 | 1.057 | -0.411 | -0.527 | -0.053 | 0.101 | 0.035 | 0.059 |
| **P3** | 1.789 | -2.670 | 0.548 | 2.571 | -0.279 | 0.046 | 0.067 | -0.112 | -0.088 | 0.054 | -0.044 |

**Table S2.** Factor coordinates of the variables, based on correlations, for the PCA results for the GC-MS data of the derivatized lipid fractions from Mangalitza hard fat (relative concentrations data for specific fatty acid methyl esters, or relative concentrations data for fatty acid methyl esters classes – SFA: saturated FAs, MUFA: monounsaturated FAs and PUFA: polyunsaturated FAs).

| Variable | Fact. 1 | Fact. 2 | Fact. 3 | Fact. 4 | Fact. 5 | Fact. 6 | Fact. 7 | Fact. 8 | Fact. 9 | Fact. 10 | Fact. 11 |
|---|---|---|---|---|---|---|---|---|---|---|---|
| **C8:0/SFA** | 0.095 | -0.308 | 0.039 | 0.940 | 0.072 | 0.039 | 0.018 | 0.060 | 0.008 | 0.017 | -0.006 |
| **C10:0/SFA** | -0.965 | -0.051 | -0.123 | 0.200 | -0.013 | 0.069 | -0.013 | -0.040 | -0.017 | -0.059 | -0.001 |
| **C12:0/SFA** | -0.994 | 0.016 | -0.064 | -0.040 | 0.004 | 0.019 | -0.030 | 0.050 | -0.036 | -0.045 | 0.005 |
| **C14:0/SFA** | -0.933 | -0.069 | 0.039 | 0.065 | 0.312 | -0.096 | -0.102 | 0.023 | -0.003 | -0.030 | 0.008 |
| **C15:0/SFA** | -0.939 | -0.214 | -0.128 | -0.181 | -0.094 | 0.110 | 0.032 | 0.021 | 0.016 | -0.020 | -0.037 |
| **C16:1/MUFA** | -0.835 | -0.468 | -0.007 | -0.080 | 0.271 | 0.034 | 0.037 | -0.005 | 0.004 | 0.021 | -0.004 |
| **C16:0/SFA** | 0.124 | 0.870 | -0.411 | -0.057 | 0.169 | 0.083 | 0.054 | 0.079 | 0.099 | -0.015 | 0.009 |
| **cyC16:0/cySFA** | -0.879 | -0.418 | 0.009 | -0.158 | 0.072 | 0.018 | 0.093 | 0.089 | -0.023 | 0.070 | 0.008 |
| **C17:0/SFA** | -0.962 | 0.078 | -0.165 | -0.018 | -0.194 | 0.040 | -0.012 | 0.009 | -0.029 | 0.026 | 0.018 |
| **C18:2/PUFA** | 0.571 | -0.748 | -0.314 | -0.073 | -0.032 | -0.003 | -0.088 | -0.003 | 0.025 | 0.019 | -0.022 |
| **C18:1/MUFA** | -0.393 | 0.573 | 0.691 | 0.081 | 0.053 | 0.112 | 0.027 | -0.131 | 0.004 | 0.020 | -0.004 |
| **C18:1(t)/MUFA** | 0.872 | -0.374 | 0.243 | -0.061 | 0.036 | -0.010 | 0.165 | 0.052 | -0.046 | -0.059 | 0.000 |
| **C18:0/SFA** | -0.187 | 0.891 | -0.194 | 0.145 | -0.322 | -0.027 | -0.008 | 0.070 | -0.047 | 0.011 | -0.011 |
| **C20:4/PUFA** | -0.889 | 0.183 | -0.301 | 0.135 | -0.030 | -0.204 | 0.122 | -0.092 | 0.031 | 0.003 | -0.011 |
| **C20:2/PUFA** | -0.400 | -0.814 | 0.132 | 0.060 | -0.385 | 0.016 | 0.017 | -0.017 | 0.073 | -0.021 | 0.021 |
| **C20:1/MUFA** | -0.456 | 0.175 | 0.851 | -0.081 | -0.078 | -0.092 | -0.035 | 0.113 | 0.048 | -0.003 | -0.012 |

**Table S3.** Eigenvalues of correlation matrix, and related statistics, for the PCA results for the GC-MS data of the derivatized lipid fractions from Mangalitza hard fat (relative concentrations data for specific fatty acid methyl esters, or relative concentrations data for fatty acid methyl esters classes – SFA: saturated FAs, MUFA: monounsaturated FAs and PUFA: polyunsaturated FAs).

| Active variable | Eigenvalue | % Total variance | Cumulative Eigenvalue | Cumulative % |
|---|---|---|---|---|
| **Var. 1** | 8.5255 | 53.2842 | 8.5255 | 53.2842 |
| **Var. 2** | 3.8551 | 24.0944 | 12.3806 | 77.3787 |
| **Var. 3** | 1.7392 | 10.8699 | 14.1198 | 88.2486 |
| **Var. 4** | 1.0620 | 6.6376 | 15.1818 | 94.8862 |
| **Var. 5** | 0.5210 | 3.2561 | 15.7028 | 98.1423 |
| **Var. 6** | 0.1017 | 0.6358 | 15.8045 | 98.7781 |
| **Var. 7** | 0.0780 | 0.4875 | 15.8825 | 99.2656 |
| **Var. 8** | 0.0692 | 0.4324 | 15.9517 | 99.6980 |
| **Var. 9** | 0.0268 | 0.1677 | 15.9785 | 99.8657 |
| **Var. 10** | 0.0182 | 0.1138 | 15.9967 | 99.9794 |
| **Var. 11** | 0.0033 | 0.0206 | 16.0000 | 100.0000 |

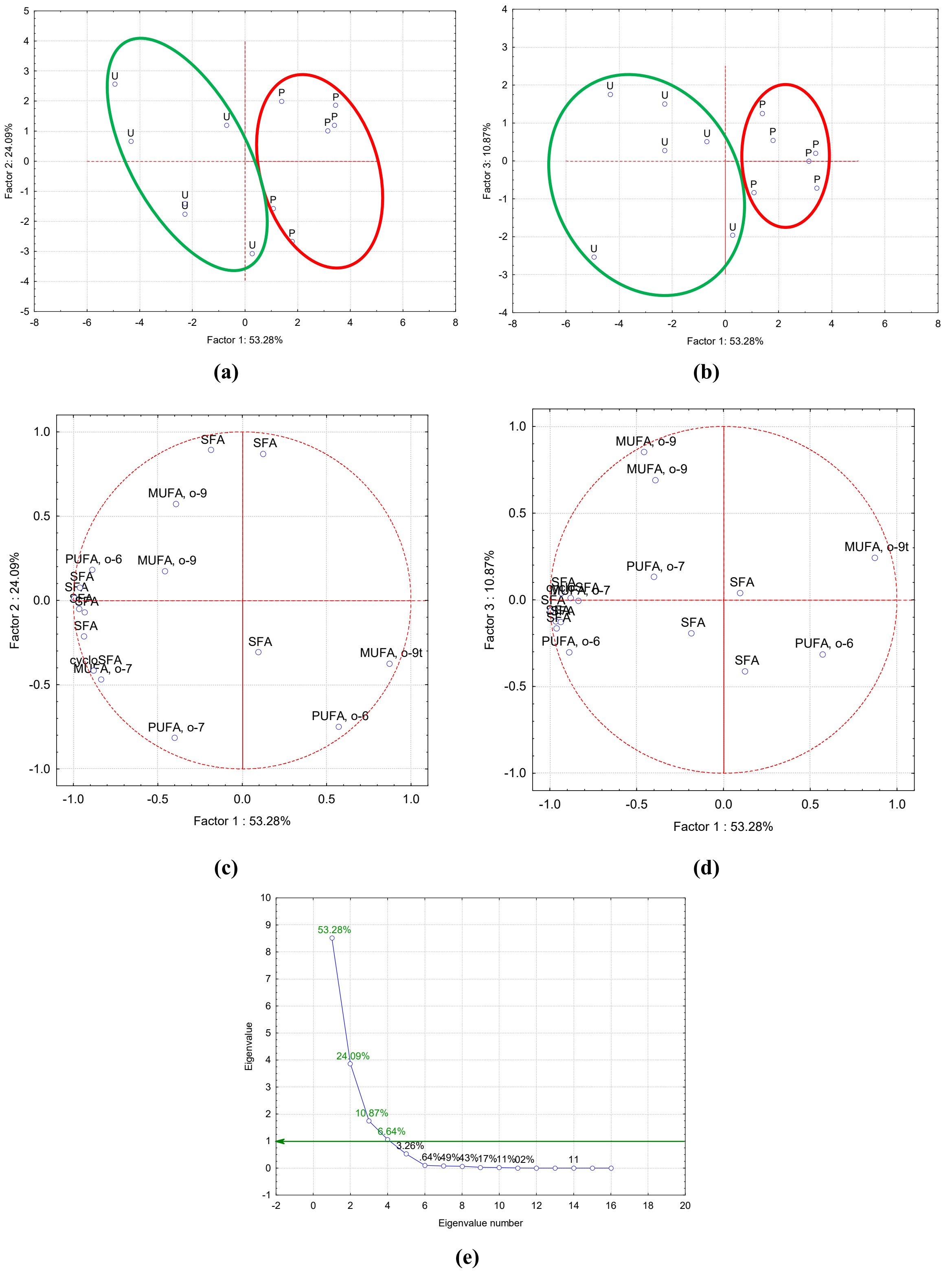


**Figure S13.** PCA results for the GC-MS data of the derivatized lipid fractions from Mangalitza hard fat (relative concentrations data for fatty acid methyl esters class – SFA: saturated FAs, MUFA: monounsaturated FAs and PUFA: polyunsaturated FAs; o-6/7/9 - ω class, *t* – stands for *trans*): (**a**) $PC_2$ *versus* $PC_1$ scores plot; (**b**) $PC_3$ *versus* $PC_1$ scores plot; (**c**) $PC_2$ *versus* $PC_1$ loadings plot; (**d**) $PC_3$ *versus* $PC_1$ loadings plot; (**e**) Eigenvalues of the correlation matrix (PCs with significant influence to the explained variance are presented in green).

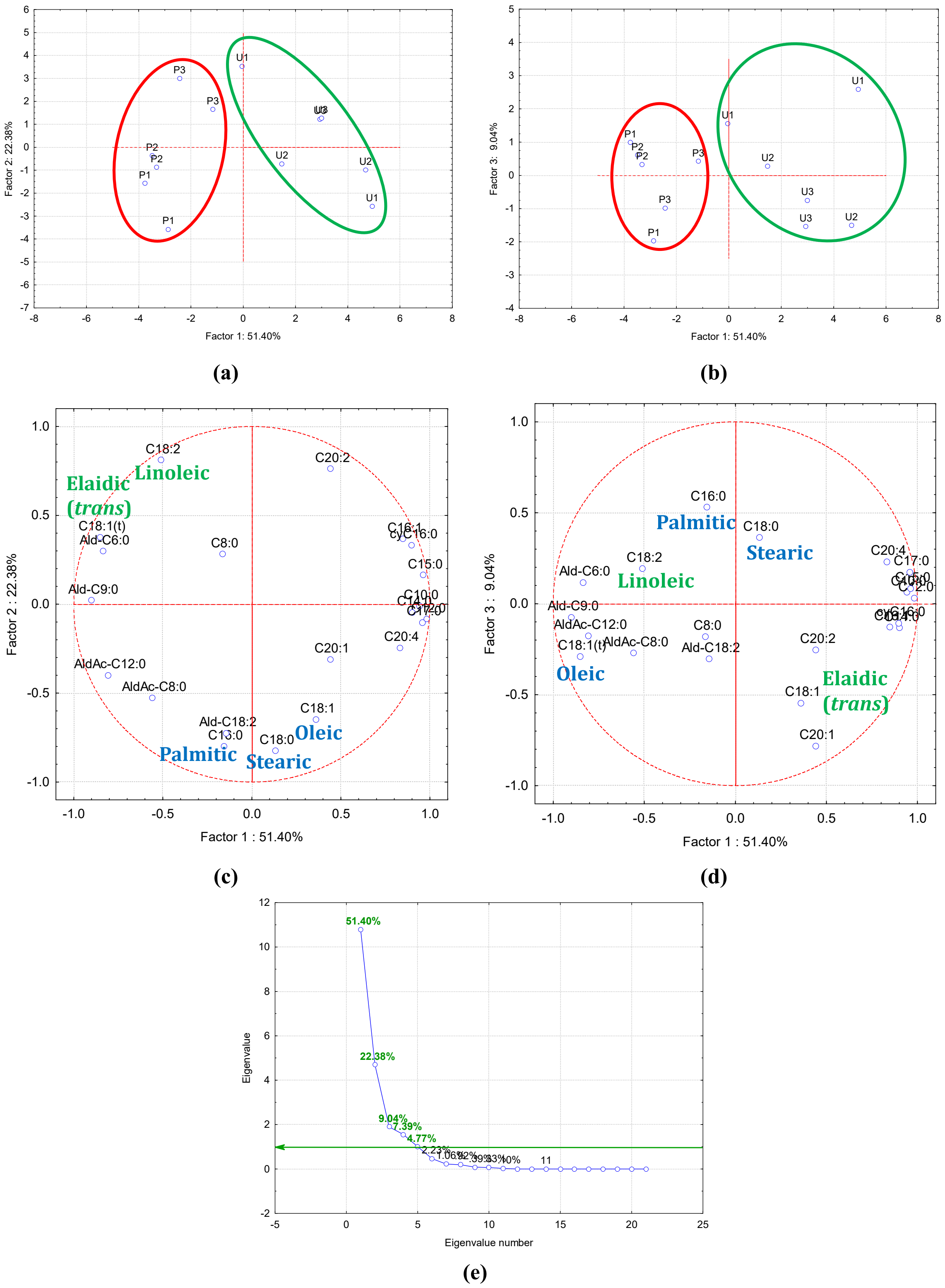


**Figure S14.** PCA results for the GC-MS data of the derivatized lipid fractions from Mangalitza hard fat (relative concentrations data for specific fatty acid methyl esters and for degradation aldehyde dimethylacetals): (**a**) $PC_2$ *versus* $PC_1$ scores plot; (**b**) $PC_3$ *versus* $PC_1$ scores plot; (**c**) $PC_2$ *versus* $PC_1$ loadings plot; (**d**) $PC_3$ *versus* $PC_1$ loadings plot; (**e**) Eigenvalues of the correlation matrix (PCs with significant influence to the explained variance are presented in green).

**Table S4.** Factor coordinates of cases, based on correlations, for the PCA results for the GC-MS data of the derivatized lipid fractions from Mangalitza hard fat – Mg_U1/2/3 and Mg_P1/2/3 (or U and P for unprocessed and processed samples), in comparison with landrace pig hard fat – Pk / P_Pk (relative concentrations data for specific fatty acid methyl esters, or relative concentrations data for fatty acid methyl esters class – SFA: saturated FAs, MUFA: monounsaturated FAs and PUFA: polyunsaturated FAs).

| Case | Fact. 1 | Fact. 2 | Fact. 3 | Fact. 4 | Fact. 5 | Fact. 6 | Fact. 7 | Fact. 8 | Fact. 9 | Fact. 10 |
|---|---|---|---|---|---|---|---|---|---|---|
| **Pk/P_Pk** | 3.947 | 4.421 | -0.342 | -0.555 | 0.170 | 0.056 | -0.026 | 0.039 | 0.004 | 0.001 |
| **Mg_U1** | -4.244 | 0.202 | -1.027 | -0.846 | 0.425 | 0.175 | 0.079 | 0.093 | -0.007 | 0.006 |
| **Mg_U1** | 0.030 | -1.272 | 2.386 | -0.933 | -0.565 | 0.125 | -0.052 | 0.044 | 0.044 | 0.003 |
| **Mg_U2** | -0.709 | -0.115 | -1.371 | -0.586 | -0.081 | -0.364 | 0.056 | -0.230 | 0.029 | -0.003 |
| **Mg_U2** | -2.682 | 2.081 | -0.696 | 0.411 | -0.512 | -0.180 | -0.112 | 0.083 | -0.050 | -0.014 |
| **Mg_U3** | -1.031 | 1.484 | 0.946 | 0.703 | -0.018 | 0.289 | 0.122 | -0.121 | 0.033 | -0.018 |
| **Mg_U3** | -1.318 | 0.891 | 1.757 | 0.599 | 0.194 | 0.105 | -0.095 | -0.081 | -0.015 | 0.028 |
| **Mg_P1** | 1.099 | -2.145 | -1.147 | 0.109 | 0.204 | 0.274 | -0.055 | -0.026 | -0.006 | 0.002 |
| **Mg_P1** | 0.602 | -0.563 | -1.766 | 0.729 | -0.185 | -0.101 | 0.034 | 0.112 | 0.081 | 0.023 |
| **Mg_P2** | 1.314 | -1.547 | -0.529 | 0.177 | -0.015 | 0.170 | 0.102 | 0.048 | -0.007 | -0.033 |
| **Mg_P2** | 1.509 | -1.549 | -0.885 | -0.073 | -0.139 | 0.084 | -0.218 | -0.063 | -0.066 | 0.008 |
| **Mg_P3** | 0.179 | -1.046 | 1.568 | 0.171 | 0.542 | -0.396 | -0.175 | 0.064 | 0.024 | -0.017 |
| **Mg_P3** | 1.305 | -0.840 | 1.107 | 0.093 | -0.020 | -0.236 | 0.341 | 0.036 | -0.065 | 0.013 |

**Table S5.** Factor coordinates of the variables, based on correlations, for the PCA results for the GC-MS data of the derivatized lipid fractions from Mangalitza hard fat – Mg_U1/2/3 and Mg_P1/2/3 (or U and P for unprocessed and processed samples), in comparison with landrace pig hard fat – Pk / P_Pk (relative concentrations data for specific fatty acid methyl esters, or relative concentrations data for fatty acid methyl esters class – SFA: saturated FAs, MUFA: monounsaturated FAs and PUFA: polyunsaturated FAs).

| Variable | Fact. 1 | Fact. 2 | Fact. 3 | Fact. 4 | Fact. 5 | Fact. 6 | Fact. 7 | Fact. 8 | Fact. 9 | Fact. 10 |
|---|---|---|---|---|---|---|---|---|---|---|
| **C14:0/SFA** | -0.918 | -0.140 | 0.178 | 0.299 | 0.022 | -0.124 | 0.023 | 0.012 | -0.003 | -0.007 |
| **C16:1/MUFA** | -0.703 | 0.379 | 0.576 | 0.159 | 0.059 | 0.035 | 0.021 | -0.014 | -0.006 | 0.012 |
| **C16:0/SFA** | -0.561 | -0.766 | -0.234 | 0.170 | 0.012 | 0.081 | -0.076 | -0.047 | 0.001 | -0.002 |
| **cyC16:0/cySFA** | -0.519 | 0.689 | 0.483 | -0.049 | -0.089 | 0.100 | -0.041 | 0.028 | -0.013 | -0.007 |
| **C17:0/SFA** | -0.934 | 0.249 | 0.036 | -0.211 | -0.123 | -0.006 | 0.049 | -0.046 | 0.017 | -0.002 |
| **C18:2/PUFA** | 0.488 | -0.435 | 0.732 | -0.165 | -0.014 | -0.082 | -0.021 | -0.042 | -0.019 | -0.001 |
| **C18:1/MUFA** | 0.259 | 0.832 | -0.471 | 0.064 | 0.094 | 0.030 | 0.042 | -0.045 | -0.019 | -0.004 |
| **C18:1(t)/MUFA** | 0.858 | -0.306 | 0.231 | 0.279 | -0.177 | 0.060 | 0.056 | -0.009 | 0.001 | -0.001 |
| **C18:0/SFA** | -0.613 | -0.513 | -0.580 | -0.080 | -0.127 | -0.015 | 0.020 | 0.016 | -0.026 | 0.004 |
| **C20:1/MUFA** | 0.268 | 0.902 | -0.256 | 0.130 | -0.135 | -0.094 | -0.070 | -0.015 | 0.003 | 0.004 |

**Table S6.** Eigenvalues of correlation matrix, and related statistics, for the PCA results for the GC-MS data of the derivatized lipid fractions from Mangalitza hard fat – Mg_U1/2/3 and Mg_P1/2/3 (or U and P for unprocessed and processed samples), in comparison with landrace pig hard fat – Pk / P_Pk (relative concentrations data for specific fatty acid methyl esters, or relative concentrations data for fatty acid methyl esters class – SFA: saturated FAs, MUFA: monounsaturated FAs and PUFA: polyunsaturated FAs).

| Active variable | Eigenvalue | % Total variance | Cumulative Eigenvalue | Cumulative % |
|---|---|---|---|---|
| **Var. 1** | 4.2831 | 42.8309 | 4.2831 | 42.8309 |
| **Var. 2** | 3.3384 | 33.3835 | 7.6214 | 76.2144 |
| **Var. 3** | 1.8665 | 18.6647 | 9.4879 | 94.8791 |
| **Var. 4** | 0.3231 | 3.2306 | 9.8110 | 98.1097 |
| **Var. 5** | 0.1020 | 1.0197 | 9.9129 | 99.1294 |
| **Var. 6** | 0.0536 | 0.5358 | 9.9665 | 99.6652 |
| **Var. 7** | 0.0215 | 0.2150 | 9.9880 | 99.8802 |
| **Var. 8** | 0.0098 | 0.0982 | 9.9978 | 99.9783 |
| **Var. 9** | 0.0019 | 0.0188 | 9.9997 | 99.9971 |
| **Var. 10** | 0.0003 | 0.0029 | 10.0000 | 100.0000 |

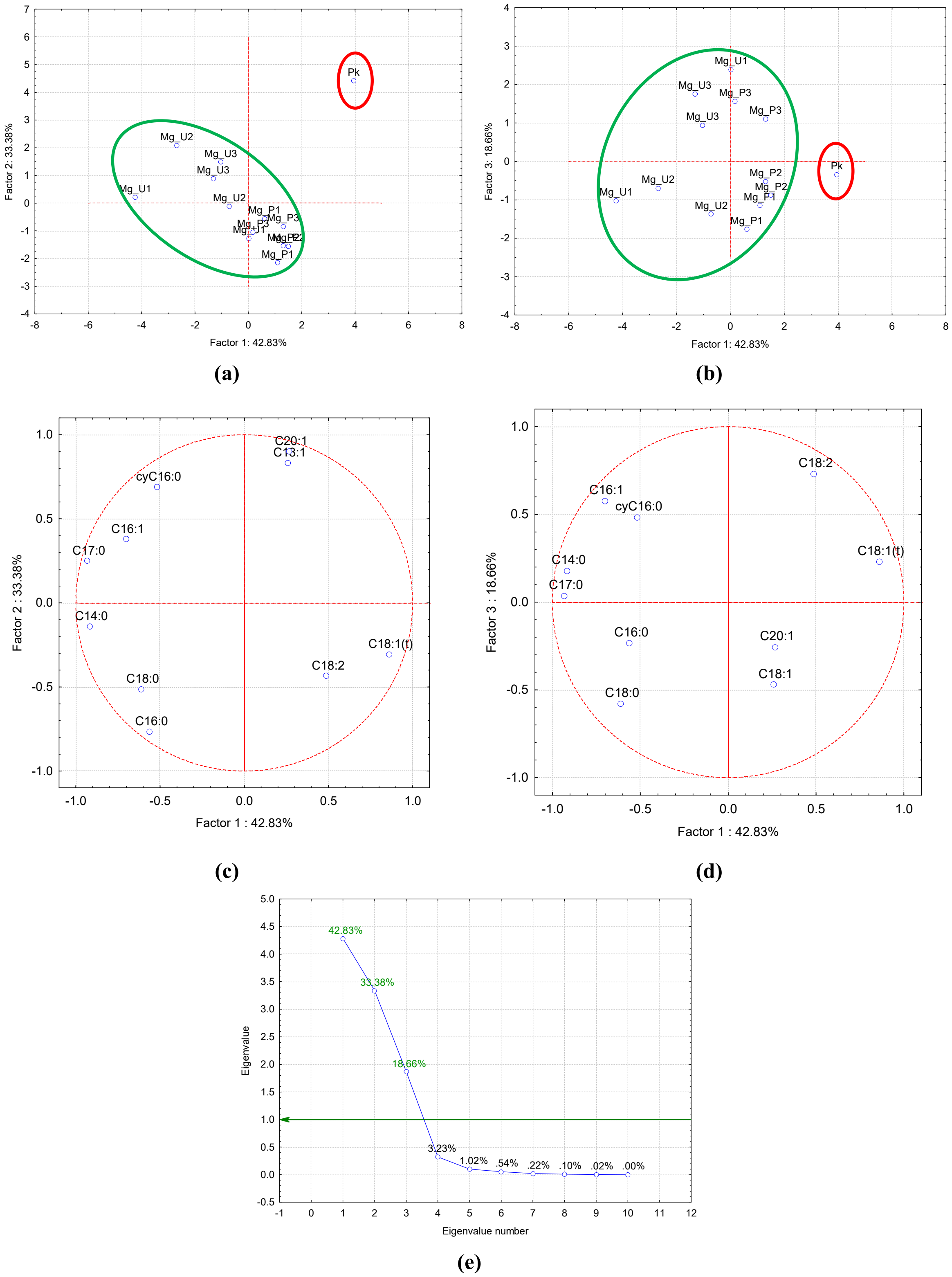


**Figure S15.** PCA results for the GC-MS data of the derivatized lipid fractions from Mangalitza hard fat – Mg_U1/2/3 and Mg_P1/2/3, in comparison with landrace pig hard fat - Pk (relative concentrations data for specific fatty acid methyl esters): (**a**) $PC_2$ *versus* $PC_1$ scores plot; (**b**) $PC_3$ *versus* $PC_1$ scores plot; (**c**) $PC_2$ *versus* $PC_1$ loadings plot; (**d**) $PC_3$ *versus* $PC_1$ loadings plot; (**e**) Eigenvalues of the correlation matrix (PCs with significant influence to the explained variance are presented in green).

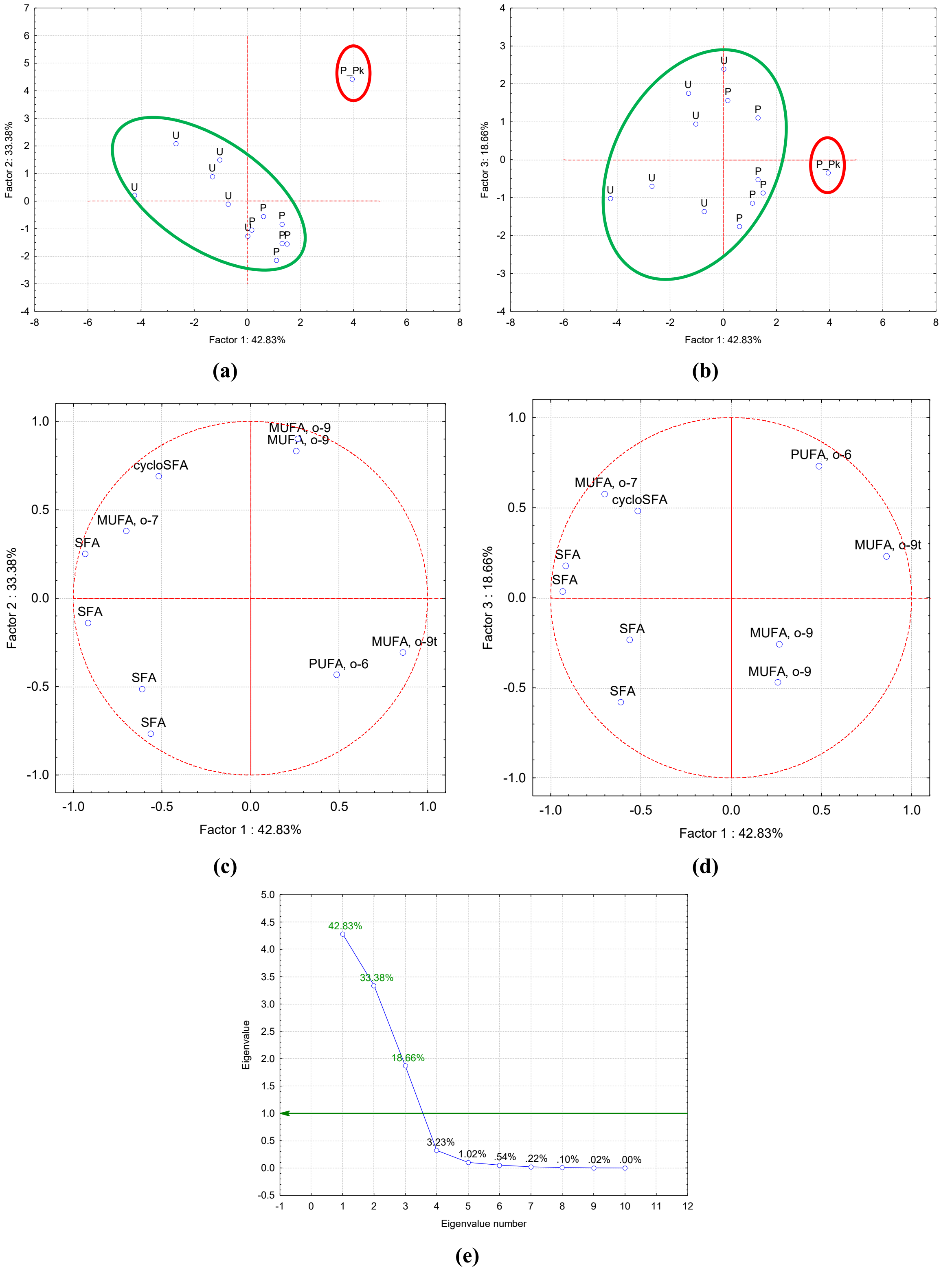


**Figure S16.** PCA results for the GC-MS data of the derivatized lipid fractions from Mangalitza hard fat – U and P, in comparison with landrace pig hard fat – P_Pk (relative concentrations data for fatty acid methyl esters class – SFA: saturated FAs, MUFA: monounsaturated FAs and PUFA: polyunsaturated FAs; o-6/7/9 - ω class, *t* – stands for *trans*): (**a**) $PC_2$ *versus* $PC_1$ scores plot; (**b**) $PC_3$ *versus* $PC_1$ scores plot; (**c**) $PC_2$ *versus* $PC_1$ loadings plot; (**d**) $PC_3$ *versus* $PC_1$ loadings plot; (**e**) Eigenvalues of the correlation matrix (PCs with significant influence to the explained variance are presented in green).
